# Vitamin B_12_ uptake across the mycobacterial outer membrane is influenced by membrane permeability in *Mycobacterium marinum*

**DOI:** 10.1128/spectrum.03168-23

**Published:** 2024-05-09

**Authors:** Beatriz Izquierdo Lafuente, Theo Verboom, Sita Coenraads, Roy Ummels, Wilbert Bitter, Alexander Speer

**Affiliations:** 1 Section Molecular Microbiology, Amsterdam Institute of Molecular and Life Sciences (AIMMS), Vrije Universiteit Amsterdam, Amsterdam, The Netherlands; 2 Department of Medical Microbiology and Infection Control, Amsterdam UMC, Amsterdam, The Netherlands; CNRS-University of Toulouse, Toulouse, France

**Keywords:** mycobacteria, vitamin B_12_, cobalamin, nutrient acquisition, cell wall, zebrafish infection

## Abstract

Vitamin B_12_ (B_12_) serves as a critical cofactor within mycobacterial metabolism. While some pathogenic strains can synthesize B_12_ *de novo*, others rely on host-acquired B_12_. In this investigation, we studied the transport of vitamin B_12_ in *Mycobacterium marinum* using B_12_-auxotrophic and B_12_-sensitive strains by deleting *metH* or *metE*, respectively. These two enzymes rely on B_12_ in different ways to function as methionine synthases. We used these strains to select mutants affecting B_12_ scavenging and confirmed their phenotypes during growth experiments *in vitro*. Our analysis of B_12_ uptake mechanisms revealed that membrane lipids and cell wall integrity play an essential role in cell envelope transport. Furthermore, we identified a potential transcription regulator that responds to B_12_. Our study demonstrates that *M. marinum* can take up exogenous B_12_ and that altering mycobacterial membrane integrity affects B_12_ uptake. Finally, during zebrafish infection using B_12_-auxotrophic and B_12_-sensitive strains, we found that B_12_ is available for virulent mycobacteria *in vivo*.

**IMPORTANCE:**

Our study investigates how mycobacteria acquire essential vitamin B_12_. These microbes, including those causing tuberculosis, face challenges in nutrient uptake due to their strong outer layer. We focused on *Mycobacterium marinum*, similar to TB bacteria, to uncover its vitamin B_12_ absorption. We used modified strains unable to produce their own B_12_ and discovered that *M. marinum* can indeed absorb it from the environment, even during infections. Changes in the outer layer composition affect this process, and genes related to membrane integrity play key roles. These findings illuminate the interaction between mycobacteria and their environment, offering insights into combatting diseases like tuberculosis through innovative strategies. Our concise research underscores the pivotal role of vitamin B_12_ in microbial survival and its potential applications in disease control.

## INTRODUCTION

Vitamin B_12_ (B_12_) is a cofactor required for core metabolic pathways in many organisms. However, despite its importance in metabolism, the synthesis of vitamin B_12_ is restricted to certain bacteria and *archaea* (1). Mycobacteria and related actinobacteria have the genetic capacity to produce this molecule *de novo* (2). The genetic repertoire necessary to synthesize B_12_ is energetically costly and contains around 25 enzymes, known as the *cob* genes family (3, 4). Nontuberculous and fast-growing mycobacteria species such as *Mycobacterium smegmatis* and *Mycobacterium abscessus* have been shown to synthesize B_12_ in culture conditions (5, 6). In contrast, the slow-growing mycobacterium species, *Mycobacterium tuberculosis* (Mtb), the pathogen responsible for the human disease tuberculosis, does not contain all genes required for *de novo* synthesis and was reported to utilize exogenous B_12_ (5, 7). *Mycobacterium leprae* is also a pathogenic mycobacterium species with a decreased genome size compared to its phylogenetic relatives. *M. leprae* lacks most of the genes involved in the B_12_ synthesis pathway and is believed to lack the ability to produce B_12_ (2). These mycobacteria species do contain B_12_-dependent proteins with distinct roles, including NrdZ (Rv0570) for DNA repair, MutAB proteins (Rv1492-1493) for fatty acid and cholesterol metabolism, and MetH (Rv2124c) involved in methionine synthesis in which B_12_ is used as a cofactor for an efficient functionality (2, 3, 8–10).

The MetH enzyme operates in the last step of the methionine biosynthesis by utilizing L-homocysteine and 5-methyltetrahydrofolate (N5-MeTHF) as substrates to produce L-methionine (2, 7). Surprisingly, mycobacteria also possess an alternative non-B_12_-dependent methionine synthase, the MetE protein (Rv1133c) (2, 10). The significant difference between these methionine synthase proteins is that, in *Escherichia coli*, MetE has a Michaelis constant value (*K*_M_) approximately 100-fold lower than MetH and, thus, presents less binding capacity toward L-homocysteine. This difference is compensated by increasing the rate of transcripts to obtain an equivalent yield of methionine synthase (11). In the presence of vitamin B_12_, methionine synthesis by MetH is preferable and energetically favorable compared to MetE. Furthermore, vitamin B_12_ regulates the gene expression of *metE* by an RNA riboswitch in the upstream region of the mRNA that blocks its translation (6). The presence of these protein pairs allows mycobacteria to adapt to different environments depending on the B_12_ availability. Notably, this connection between vitamin B_12_ and the methionine synthases MetH and MetE helped Gopinath et al. identify the uptake transporter that transfers B_12_ to the cytosol. Rv1819c was identified as the sole B_12_ cytosolic transporter under standard *in vitro* conditions in *M. tuberculosis* (12).

How B_12_ is transported across the outer membrane of mycobacteria is unknown. In gram-negative bacteria, this scavenging process across the outer membrane is carried out by the BtuB. The TonB-dependent BtuB protein is the outer membrane receptor and transporter specific for vitamin B_12_ and its derivatives with the assistance of the TonB complex. This TonB complex, composed of TonB-, ExbB, and ExbD, provides the energy necessary for substrate translocation (13, 14). However, the mycobacteria genus lacks structural homologs of this scavenging pathway.

In this study, we utilize *Mycobacterium marinum* as a surrogate to study the B_12_ scavenging mechanism of virulent human mycobacteria. Since *M. marinum* contains the known B_12_-dependent enzymes and riboswitches and is also genetically close to the species of Mtb and *M. leprae*, we considered *M. marinum* a good model for our research. Our results give us more insights into the scavenging process of vitamin B_12_ in pathogenic mycobacteria and its importance during infection.

## RESULTS

### *M. marinum* utilizes exogenous vitamin B_12_

**Fig 1:**
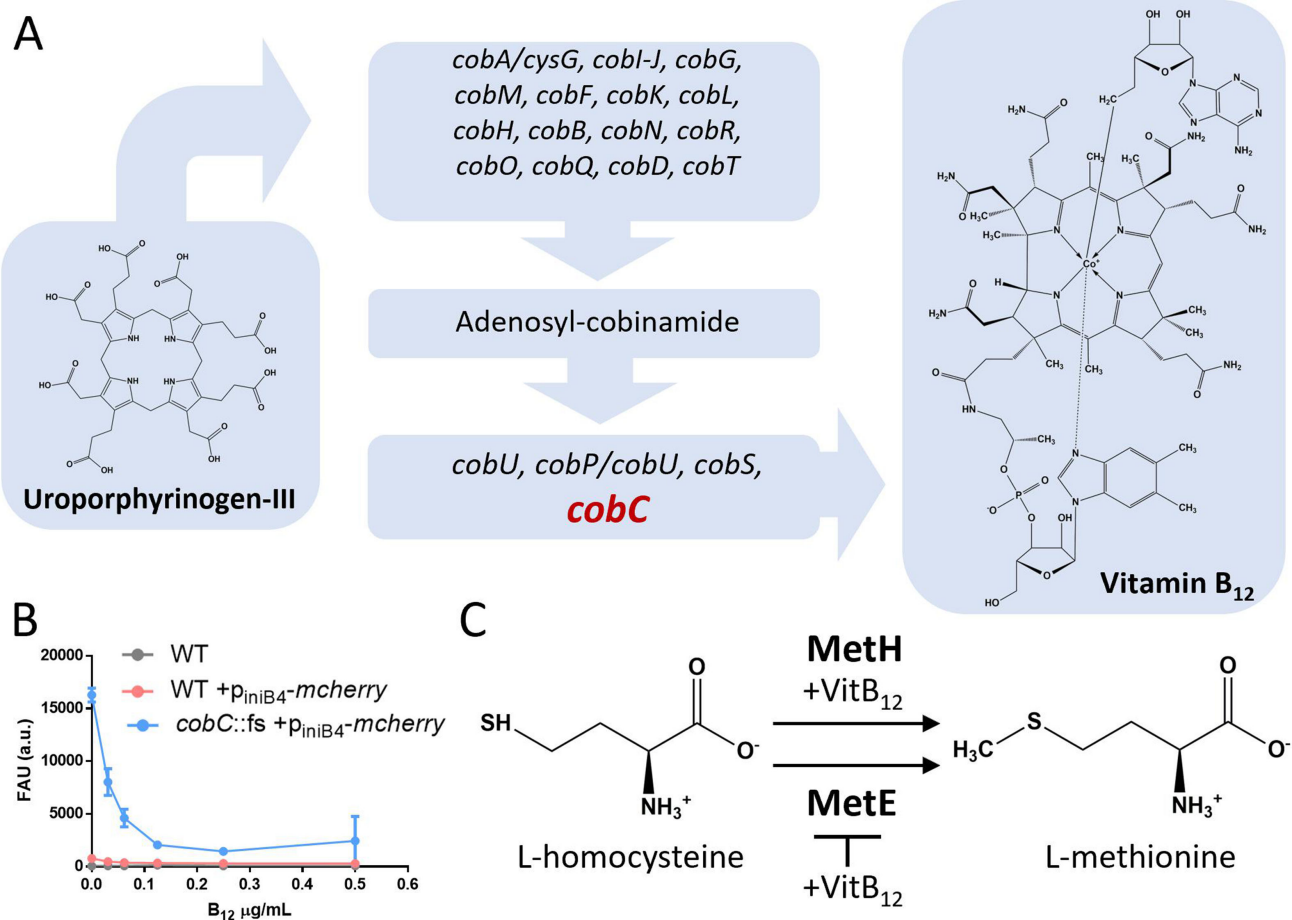
B_12_ synthesis and metabolism in mycobacteria. (A) B_12_ synthesis and metabolism in *M. marinum*; the *cobC* gene (in red) was mutated (*cobC::fs*) to disrupt B_12_ synthesis in *M. marinum M^USA^*. (**B)** Reporter assay employing a B_12_-sensitive *p_inib4_-mCherry* construct in a *cobC* mutant strain. This assay detects incomplete synthesis pathways, producing a fluorescent signal. Bacteria were exposed to varying B_12_ concentrations, leading to signal reduction with increased B_12_ levels. *M. marinum* WT (wild-type) strain and WT expressing the reporter construct were used as controls. (**C)** The enzymes MetH and MetE convert L-homocysteine into L-methionine. MetH relies on B_12_ as a cofactor, while MetE operates independently of B_12_. Translation of *metE* is regulated by an RNA riboswitch.

In contrast to *M. tuberculosis,* the genome of *M. marinum* seems to contain the entire gene repertoire, the *cob* genes family, required to produce vitamin B_12_. Previous analysis showed that these *cob* genes have an active function as disruption of any of these genes or the genes coding for vitamin B_12_-dependent MutAB strongly induces the iniBAC stress system (15). The same study also showed that *cob* genes, including aminotransferase *cobC* (*mmar_3307*), are dispensable under standard culture conditions (15, 16). To investigate the transport mechanism of vitamin B_12_ in mycobacteria, we abolished B_12_ synthesis in *M. marinum* by creating a frameshift mutation in *cobC* (*cobC::fs*) (Fig. 1A).

First, to demonstrate that the vitamin B_12_ synthesis pathway was abolished in the *cobC*::fs mutant strain, we employed the previously described iniBAC stress system, a B_12_-sensitive reporter (Fig. 1B) (15). This reporter construct (p_iniB4_-*mcherry*) contains the promoter region of the *iniBAC* operon fused to the gene *mcherry,* which is responsive to intracellular B_12_ levels in mycobacteria (15). We transformed the *M. marinum* WT (wild-type) and the *cobC* mutant strain with the reporter construct, exposed the bacteria to different concentrations of B_12_, and determined the amount of produced Mcherry by fluorescence. In the absence of exogenous B_12_, we observed no reporter activity in the WT strain, while the *cobC*::fs strain showed an elevated fluorescent signal of the reporter construct. This signal was quenched in the presence of exogenous B_12_ in a dose-dependent manner, suggesting that the *cobC*::fs strain’s ability to synthesize B_12_ was decreased or abrogated as compared to *M. marinum* WT.

**Fig 2:**
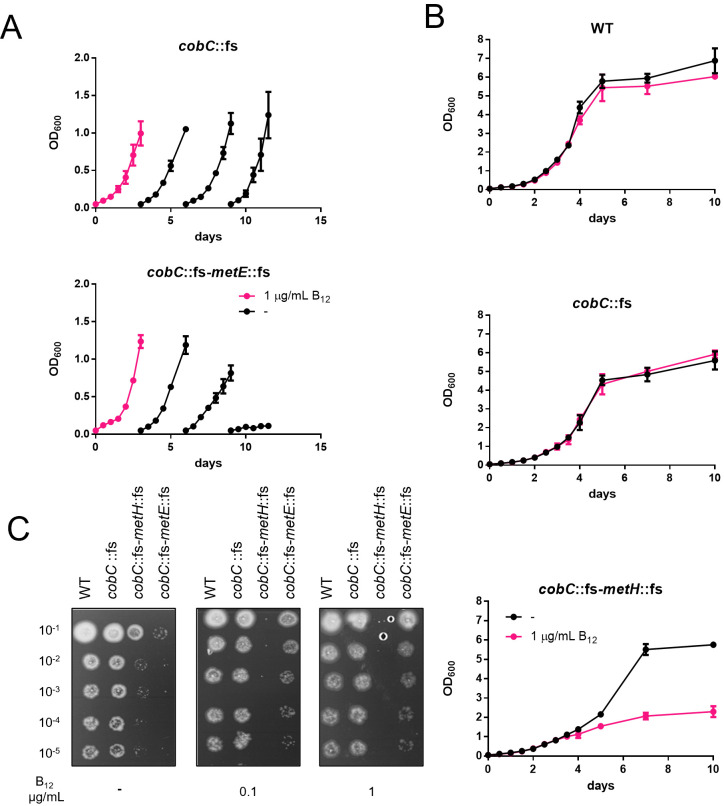
B_12_ is scavenged in *M. marinum.* (A) The *cobC-metE*::fs mutant is B_12_-auxotrophic. Bacteria were inoculated at optical density (OD_600_) 0.05 and grown with 1 µg/mL B_12_. After bacterial cultures reached the logarithmic phase (OD_600_ ~ 1), bacteria were diluted to OD_600_ 0.05 and grown in media without B_12_. The *cobC*::fs strain served as control. (**B)** Growth curves of WT, *cobC*::fs, and *cobC-metH*::fs strains. Bacteria were started at OD_600_ 0.05 and grown with and without 1 µg/mL B_12_** (C)** Growth assays in WT, *cobC*::fs, and *cobC-metH*::fs in 7H10 with and without B_12_. Bacteria in the logarithmic phase were spotted in 10-fold serial dilutions and incubated for a week until visible growth.

Deleting *metH* or *metE* results in two mutant strains with opposite B_12_-dependent growth phenotypes. In the absence of *de novo* B_12_ synthesis, the deletion of *metE* creates a B_12_-auxotrophic strain, while the deletion of *metH* prevents the bacteria from growing in the presence of B_12_ due to the repression of the *metE* gene (Fig. 1C). Within the *cobC* mutant background, we constructed individual *metH* (*mmar_4825*) or *metE* (*mmar_4328*) frameshift mutants (*cobC*::fs-*metH::fs, cobC-metH; cobC*::fs-*metE::fs, cobC-metE*) and determined their susceptibility to external B_12_ (Fig. 2).

The B_12_ auxotrophic strain *cobC*::fs*-metE*::fs was routinely grown in the presence of B_12_. No immediate growth defect was observed after omitting B_12_ from the medium (Fig. 2A). B_12_ is a micronutrient, and the remaining B_12_ in the cells appears to be enough to support growth for several generations. To determine when a growth defect can be observed, we monitored the growth of the *cobC-metE* mutant after B_12_ starvation. The *cobC* mutant parent strain served as a control. Bacteria were first grown in the presence of B_12_. After the control strain reached an OD_600_ of approximately 1.0, the strains were reinoculated in a fresh medium without B_12_. The first two growth curve experiments observed no growth defect of the *cobC-metE* mutant. After the third reinoculation in B_12_-free media, we could detect abrogated growth of the *cobC-metE* mutant, probably caused by the essentiality of exogenous B_12_. This auxotrophic phenotype could be chemically complemented when the *cobC-metE* mutant was grown in media containing methionine, indicating that B_12_ auxotrophy was indeed caused by the lack of B_12_-dependent methionine production by MetH (Fig. 1B; Fig. S1).

Likewise, we tested the *cobC-metH* mutant. The bacterial strains were cultured in a medium containing no B_12_ or 1 µg/mL of B_12_, and growth was measured over time (Fig. 2B). Bacterial cultures were started at OD_600_ 0.05, and growth was monitored every 12–24 h for several days. The WT and *cobC* mutant strains showed no growth difference with or without the vitamin, whereas the *cobC-metH* mutant stopped replicating at an OD_600_ of around 1 in the B_12_-supplemented medium. Also, this phenotype, observed for the *cobC-metH* mutant, could be chemically complemented by adding methionine to the media (Fig. S2).

Comparable phenotypes were observed when the strains were grown on solid media in the presence or absence of B_12_. The *cobC*::fs-*metH:*:fs and *cobC*::fs-*metE*::fs mutant and control strains WT and *cobC* mutant were spotted in serial dilutions on 7H10 agar plates supplemented with increasing concentrations of B_12_ (0 µg/mL, 0.1 µg/mL, and 1 µg/mL) (Fig. 2C). The *cobC-metH* mutant presented the same drastic phenotype as observed in liquid media; no growth was detected at 1 µg/mL. The *cobC-metE::fs* mutant showed no growth without B_12_ and grew similarly to the control strains in the presence of B_12_.

Together, these results show that the constructed *M. marinum* strains can scavenge exogenous B_12_ from liquid and solid media and can be employed to study the uptake mechanisms. As an additional validation of our auxotrophic strain, we genetically complemented the *cobC-metH* mutant by expressing the wild-type copy of Mtb *metH* (*rv2124c*) and observed restored growth on B_12_-containing agar plates (Fig. S3).

### Vitamin B_12_ is available during infection in the zebrafish model

Nutrient deprivation is a known strategy of the host to limit pathogen growth and control the infection (17). Our growth experiments with exogenous B_12_ confirm that virulent mycobacteria can take up B_12_ across the cell envelope, as previously described (7). However, we aimed to investigate whether pathogenic mycobacteria gain access to B_12_ during infection.

**Fig 3:**
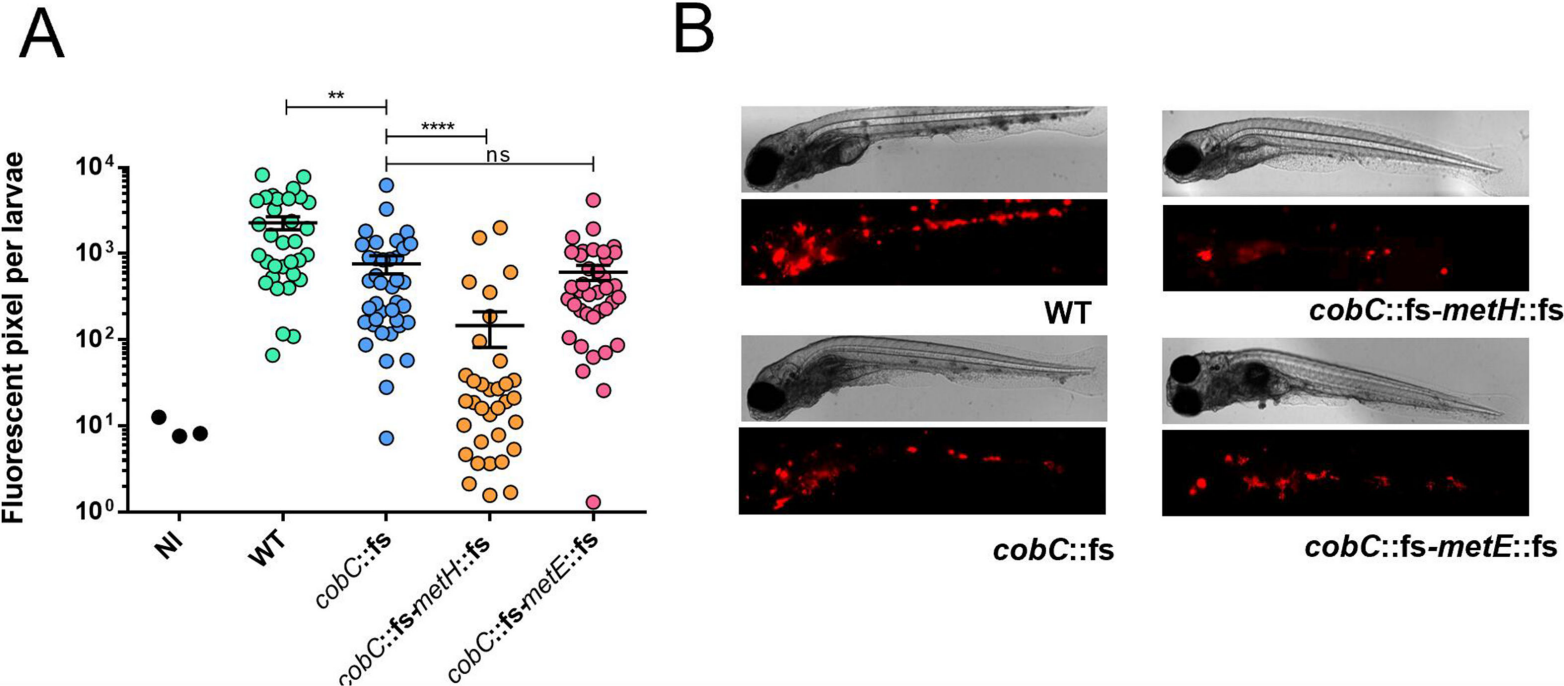
B_12_ is available to *M. marinum* during infection of zebrafish. Systemic infection of zebrafish embryo with *M. marinum* was established by injection of 50–200 CFU bacteria into the caudal vein. All bacterial strains carry a plasmid expressing RFP (*tdtomato*). The embryos were imaged at 4 days post-infection. (**A)** The level of infection was quantified by counting the pixels of red fluorescent signal per embryo. Strains: by WT, *cobC*::fs, *cobC*::fs-*metH*::fs, and *cobC*::fs-*metE*::fs; NI, noninfected. Significant changes in each group were determined by ANOVA testing. *P* > 0.05, not significant, not indicated. ^*^P < 0.05; ^**^P < 0.01; ^***^P < 0.001. (**B)** Representative pictures of the infected embryos; red pixels correspond to bacteria.

To investigate this, we used the zebrafish infection model and determined the virulence of our *cobC-metH*/*cobC-metE* mutant pair (Fig. 3A and B). Zebrafish embryos were infected by injection into the caudal vein with *M. marinum* WT or the mutant strains *cobC*, *cobC-metH,* or *cobC-metE*. All strains carried a plasmid expressing the fluorescent protein *tdtomato,* and the infection was quantified 5 days post-infection by fluorescent microscopy. Infection with the *cobC* mutant strain resulted in a modest but significantly lower bacterial burden than the WT strain, indicating that the acquisition of exogenous vitamin B_12_ in the host is a limiting factor. Interestingly, while the *cobC-metE* mutant displayed a bacterial burden comparable to the *cobC* parent strain, the *cobC-metH* mutant displayed a strongly attenuated phenotype. This result demonstrates that B_12_ is available to the bacteria during infection in the zebrafish embryo.

### Mutations in genes involved in lipid synthesis improved B_12_ uptake

Vitamin B_12_ is a large, water-soluble molecule actively transported across the cell envelope in many bacteria (18–20). To identify proteins involved in B_12_ scavenging in mycobacteria, we conducted an evolution experiment to select bacteria with increased B_12_ uptake characteristics. To this end, we generated a *cobC-metE* mutant in the background of a hypermutating strain, the *nucS* deletion strain (Δ*nucS*). The *nucS* gene encodes for an endonuclease specific for DNA mismatch repair. Inactivation of the *nucS* gene increases the emergence of spontaneous mutations by more than 20-fold, generating a hypermutable phenotype in *M. smegmatis* and *M. abscessus* (21, 22). In our experiment, the Δ*nucS-cobC*::fs*-metE*::fs (*nucS-cobC-metE*) mutant strain is auxotrophic for B_12_ and was used to select for mutations in genes that result in faster growth caused by enhanced B_12_ uptake.

**Fig 4:**
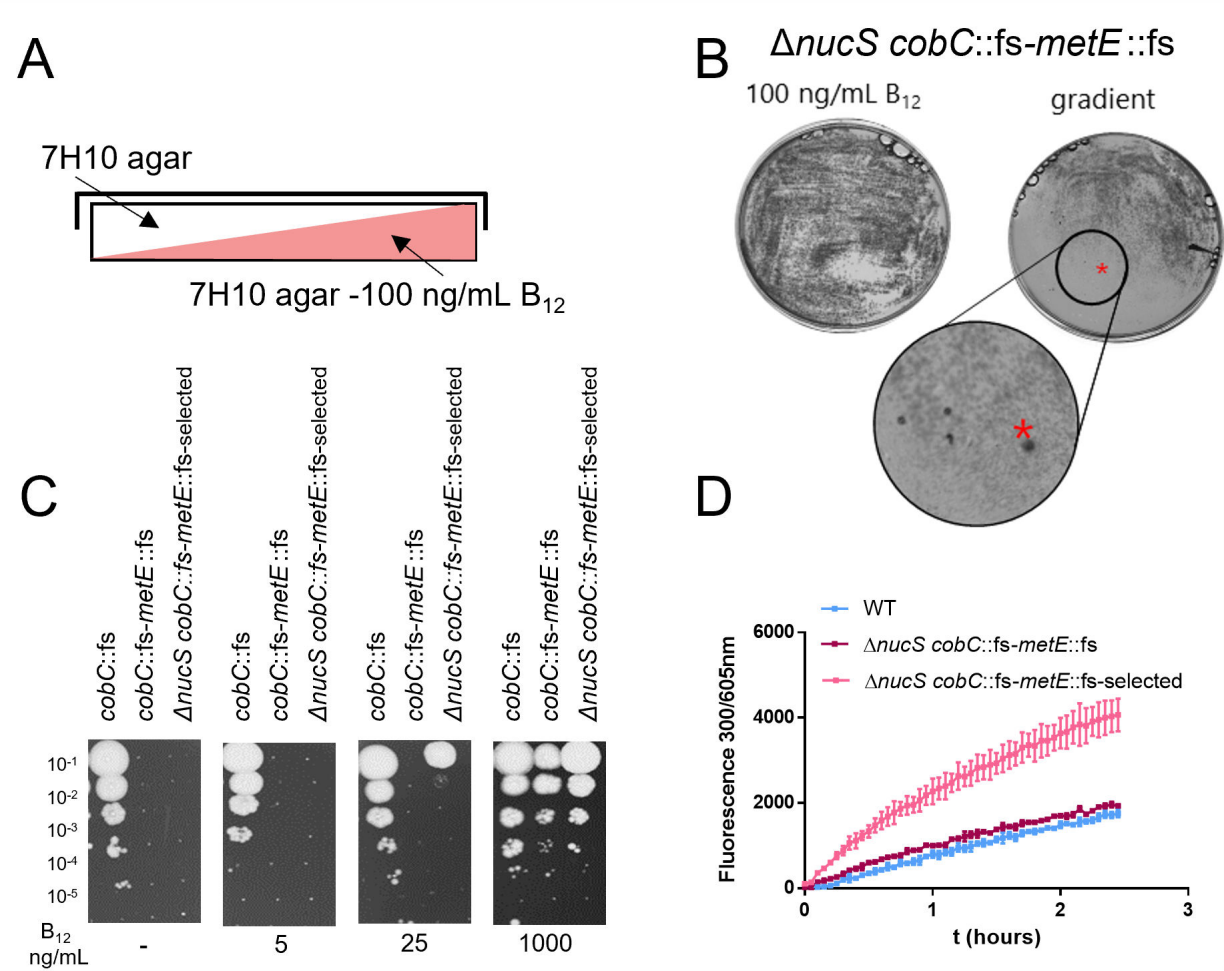
Increased B_12_ uptake capacity in the hypermutating *nucS-cobC-metE*_parental_ mutant. (A) Gradient plates containing 100 ng/mL B_12_ on 7H10 agar plates. (**B)** Selected colonies growing on regular and gradient plates containing 100 ng/mL B_12_. The marked colony (red asterisk), named Δ*nucS cobC-metE*_-selected_, was selected for further analysis. (**C)** Drop assays of bacteria growing at 0, 5, 25, and 1,000 ng/mL B_12_. Mutant strains *cobC* and *cobC-metE* served as controls. (**D)** Ethidium bromide (EtBr) uptake experiment to determine membrane permeability. Bacteria were exposed to 10 µg/mL of EtBr, and fluorescence was measured every 3 minutes (ex: 300 nm/em: 605 nm). The *nucS-cobC-metE*-_selected_ mutant shows higher EtBr uptake compared to the WT and the parental control strains.

The mutant strain *nucS-cobC-metE* was selected on agar plates with low levels of B_12_ to generate a specific selective pressure. The selection was carried out on gradient plates ranging from no B_12_ to 100 ng/mL (Fig. 4A). On these plates, more colonies were observed at the highest B_12_ levels, and the number of colonies decreased at lower concentrations. Interestingly, several single colonies with a significantly bigger size compared to the surrounding colonies grew at intermediate B_12_ concentration. The biggest colony was chosen for further analysis, *nucS-cobC-metE*-_selected_ (Fig. 4B). While the parental *nucS-cobC-metE* mutant strain could not grow at 25 ng/mL B_12_, the selected candidate, the *nucS-cobC-metE*-_selected_ mutant already displayed modest growth from 25 ng/mL (Fig. 4C). Chromosomal DNA extraction followed by whole-genome sequencing analysis was performed for the parental strain *nucS-cobC-metE* mutant and the selected colony to identify mutations linked to this improved growth survival at low B_12_ (Table 1).

**TABLE 1:** Whole genome sequencing results of *nucS-cobC-metE-*_selected_*^a^*

Gene ID	Gene name	Ortholog H37Rv	Type	Reference	Allele	Nucleotide change	Amino acid change
*mmar_0099*	*nrp*	–	SNV	T	C	2355T>C	Ile785Ile
*mmar_0099*	*nrp*	–	SNV	T	C	5565T>C	His1855His
*mmar_1774*	*ppsC*	*rv2933*	SNV	G	A	4432G>A	Gly1478*
*mmar_3097*	*nrp*	–	SNV	C	A	3387C>A	Leu1129Phe
*mmar_3098*	*pks*	–	SNV	A	T	803A>T	Asp268Val
*mmar_5044*	*pe-pgrs*	–	SNV	A	T	2546A>T	Gly849Gly
*mmar_5044*	*pe-pgrs*	–	MNV	AGC	CCG	2553AGC>CCG	Ala850Arg

^*a*^ Chromosomal DNA extraction was performed on the *nucS-cobC-metE*-_selected_ colony and further analyzed by whole-genome sequencing analysis. The strain was analyzed for variations and polymorphisms. Results are derived from comparing the strain *nucS-cobC-metE-*_parental_ with *nucS-cobC-metE-*_selected_. SNV, single-nucleotide variant. Asterisk inidcates a stop codon.

We compared the genomes of the *nucS-cobC-metE* strain (before selection) to the reference genome of *M. marinum* WT and identified several mutations. During the introduction of the *cobC* and *metE* deletion into the Δ*nucS* background, the bacteria were grown with a B_12_-supplemented medium to avoid selective pressure. The mutations that were already present in the parent strain were, therefore, neglected (Table S1). We identified seven unique mutations in the *nucS-cobC-metE*-_selected_ strain that presented improved growth on media with low B_12_ compared to its parent strain (Table 1). Mutations in *mmar_0099*, encoding for a nonribosomal peptide synthase (NRPs), were silent and did not alter the gene product. Several mutations were identified in genes involved in cell envelope biogenesis. The gene *mmar_3097*, which was already mutated (Tyr853Phe) in the parental *nucS-cobC-metE* mutant strain, contained an additional mutation causing an amino acid exchange in position Leu1129Phe. In the same operon, gene *mmar_3098* carried a mutation resulting in a missense mutation (Asp268Val). The gene *mmar_3097* encodes for an NRP, and the gene *mmar_3098* for a polyketide synthase (PKS). PKS and NRPs are enzymes that synthesize a different range of particular secondary metabolites, the so-called polyketides, and nonribosomal peptides, respectively. The majority of the PKS and NRP synthase have been linked to the synthesis of unique lipid components of the membrane of mycobacteria (23–25). The Mmar_3098 has 53% identity to PKS7 from *M. marinum*, associated with the formation of methyl-branched unsaturated membrane lipids (23). An additional mutation was found in the gene *ppsC* (*mmar_1774*), resulting in a frameshift after position Gly1478*. The PpsC protein belongs to the phthiocerol dimycocerosate (PDIM) biosynthesis pathway. PDIMs are directly associated with the membrane permeability of mycobacteria (26).

These results indicate a link between improved B_12_ uptake and changes in the membrane permeability of mycobacteria. To confirm this, we assessed changes in membrane permeability by performing EtBr accumulation assays (Fig. 4D). Briefly after the dye enters the bacteria’s cytosol and intercalates with the DNA, EtBr emits a strong fluorescent signal. Using this approach, we tested the *nucS-cobC-metE*-_selected_ mutant and compared the uptake rate of EtBr to WT and the *nucS-cobC-metE* parental strain. The *nucS-cobC-metE*-_selected_ mutant accumulated faster EtBr, and the fluorescent signal was significantly higher over time when compared to the WT but also with the parental strain, indicating that the mutations were linked to higher membrane permeability.

We repeated the assay to establish how affected the selected mutant’s membrane was. We included extra control strains with known increased cell envelope permeability as a reference, two strains with mutations in genes involved in the PDIM synthesis pathway *ppsE* (*mmar_1772*) and *papA5* (*mmar_1768*), and a *M. marinum* WT strain expressing the outer membrane protein MspA of *M. smegmatis* (Fig. S4) (27–29). As observed, all control strains accumulated EtBr as fast as the *nucS-cobC-metE*-_selected_ mutant, determining that the mutant has indeed a severe alteration on the membrane permeability status. We aimed to identify a *nucS-cobC-metE* strain capable of improved growth at low B_12_ concentrations while maintaining cell wall integrity. However, upon assessing three additional *nucS-cobC-metE* strains from the same screen, we observed increased cell wall permeability toward EtBr (Fig. S5). We acknowledge that while this approach is conceptually elegant, it proved unsuccessful due to the inclusion of PDIM-negative strains as positive hits in the screen. Loss of PDIMs under standard culture conditions is already a problem for work with all virulent mycobacteria and exacerbated in a hypermutating Δ*nucS* strain, especially when selecting for such mutations, as in our experiments.

### Deletions of genes involved in cell wall processes cause resistance to B_12_ in the *cobC-metH* mutant background

We employed a complementary assay using the *cobC-metH* mutant to identify proteins involved in B_12_ transport. This mutant cannot grow in the presence of vitamin B_12_. Using transposon mutagenesis, we screened for transposon insertions that could reverse the growth defect and allow the *cobC-metH* mutant to survive at B_12_ levels up to 1 µg/mL (MIC value in the *cobC-metH* mutant is 100 ng/mL) (Table 2).

**TABLE 2:** Identified transposon insertion sites in *cobC*::fs-*metH*::fs B_12_-resistant strains^*a*^

Gene ID	Gene product	H37Rv ortholog	Gene length	Transposon position(s) within gene (bp from 5′ of gene)
*mmar_0009*	MerR-like		762	667(3),671(2)
*mmar_0407*	Conserved		462	459
*mmar_1104*	EsxU	Rv3445c	354	–84,–43,1(2)
*mmar_2959*	Zinc-dependent alcohol dehydrogenase		1,029	237(2)
*mmar_3798*	PKS-I		16,488	1503(7),2234
*mmar_4541*	Magnesium chelatase	Rv0958	1,392	−49

^*a*^ Only transposon strains able to grow at B_12_ levels 10-fold higher than the parental *cobC*::fs-*metH*::fs strain are listed. Transposon insertions were identified by ligation-mediated PCR.

We determined the insertion site of 21 different transposon mutants. We identified hits related to metal-dependent proteins, such as the magnesium chelatase Mmar_4541 and the zinc-dependent dehydrogenase Mmar_2959. Since we were primarily interested in B_12_ transport, we focused on mutants that could play a direct role in cell wall processes. Three times, we identified the transposon insertion site in the promoter region of the *esxUT* operon, which codes for two substrates of the ESX-4 secretion system. In addition, the gene *mmar_3798*, encoding for a type I polyketide synthase (PKS-I) was prominently identified eight times in two independent transposon insertion sites. Also, one hypothetical protein was part of the candidate list. The conserved Mmar_0407 protein is described as a lipid transporter based on around a 90% similarity from *Mycobacterium ulcerans*.

In agreement with previous results, this transposon mutagenesis indicates that, membrane permeability could once again influence B_12_ transport across the outer membrane. Many of the mutant candidates had affected lipid synthesis or metabolism genes. Differences in the outer membrane composition could, thus, compromise the uptake of big and hydrophilic molecules such as B_12_.

To distinguish between cell wall changes resulting in a general lower cell wall permeability and potential specific B_12_ importers, we generated gene knockdown mutants in *M. marinum* WT and performed EtBr uptake experiments. We generated knockdowns of genes *mmar_0009, mmar_0407, mmar_1104, mmar_2959*, *mmar_3798*, and *mmar_4541*; using previously published CRISPR interference technology, WT expressing the CRISPR plasmid without any small guide RNAs (sgRNAs) served as a control (30). Interestingly, the knockdown of all selected genes resulted in decreased dye uptake compared to WT, indicating reduced cell wall permeability, except for KDmmar_0407, which showed a more similar uptake to WT (Fig. S6).

### A potential transcription regulator that could be involved in B_12_ transport

**Fig 5:**
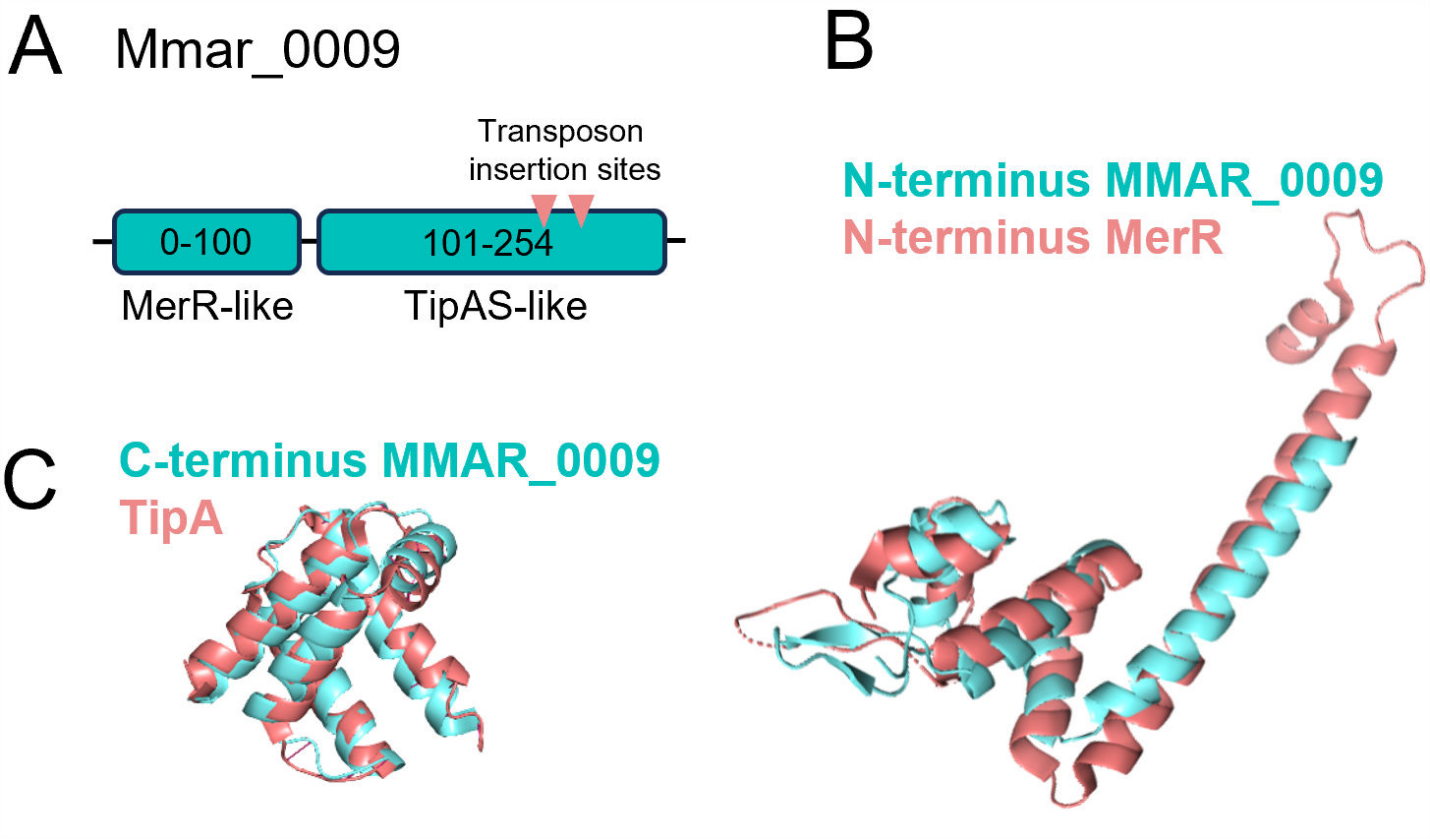
Mmar_0009 encodes for a transcriptional regulator. (A) Protein domain organization of Mmar_0009. The structure homology was predicted by PhyreR2 software and defines two domains. The N-terminal domain is predicted to be MerR-like, belonging to the transcriptional regulator family. The C-terminal domain is predicted as a TipAS-like domain, a type of MerR regulator that responds to thiopeptides. (**B)** and (**C)** Structure modeling by Alphafold prediction and structural alignments; in (**B)**, N-terminus Mmar_0009 and N-terminus MerR from *Bacillus megaterium* (PDB: 4UA1, RMSD 4.756); in (**C)**, C-terminus domain of Mmar_0009 compared with TipA domain of *Streptomyces lividans* (PDB: 1NY9, RMSD values of 1.534).

A prominent gene identified in this screen is *mmar_0009,* identified five times with two independent TA insertion sites. Protein homology studies described this gene as a MerR-like protein in mycobacteria (>80% identity, 100% coverage). Members of the MerR family are a group of transcription regulators with similar characteristics in the DNA-binding domain in the N-terminus. They present the sensor binding domain in the C-terminus (Fig. 5A) (31). This group of regulators responds to stimuli such as antibiotics, heavy metals, oxidative stress, and metal ions. The Alphafold predicted structure of the N-terminus (0–100 amino acids) of Mmar_0009 was close to the crystal structure of the N-terminus of MerR from *Bacillus megaterium* [PDB: 4UA1, root mean square deviation (RMSD) 4.756], confirming the protein homology analysis (Fig. 5B). When analyzing the structure of the Mmar_0009 sensor-binding domain, we discovered that it was structurally close to the TipAs domain found in TipAL protein, a member of the MerR family of regulators based on protein homology analysis and structural homology with Phyre2 software (54%, 100% coverage). The two transposon insertions were located within this region. The C-terminal domain of Mmar_0009’s predicted structure (100–254 amino acids, Alphafold) was compared with the TipA domain of *Streptomyces lividans* (PDB: 1NY9, RMSD values of 1.534), and both structures aligned (Fig. 5C). Thiopeptides are antibiotics formed by a macrocyclic core ring that resembles the porphyrin macrocycle found in molecules such as heme but also vitamin B_12_ (32, 33). It is possible that the Mmar_0009 protein could indeed be activated by the presence of vitamin B_12_.

### The Mmar_0009 regulon

To study the role of *mmar_0009* in the B_12_-related phenotype, we created frameshift mutants in the *cobC-metH* mutant background. Two independent mutants were then tested in growth assays on solid media to confirm the B_12_-resistant phenotype observed in the transposon mutant (Fig. S7). Indeed, the two frameshift mutants of *mmar_0009* in the *cobC-metH* background showed a significantly increased resistance to B_12_ compared to the parental *cobC-metH* mutant strain, which could be genetically complemented by the expression of an episomal copy of *mmar_0009* (Fig. S3).

We decided to map the transcriptional differences in the mutant strain by conducting RNA sequencing experiments in order to identify genes regulated by this potential identified transcription regulator. We opted to investigate the impact of *mmar_0009* on the transcriptomic profile in the *cobC-metH-mmar_0009* mutant and compare the RNA levels to the *cobC-metH* parent strain. We hypothesized that B_12_ levels could influence *mmar_0009* expression and activation in the media and, therefore, supplemented the medium with B_12_. In addition, to remove the growth-suppressing effects that B_12_ has on the *cobC-metH* strain, we additionally supplemented the medium with methionine. Bacterial RNA was extracted in the mid-logarithmic phase, and RNA sequencing was performed by parallel sequencing.

**Fig 6:**
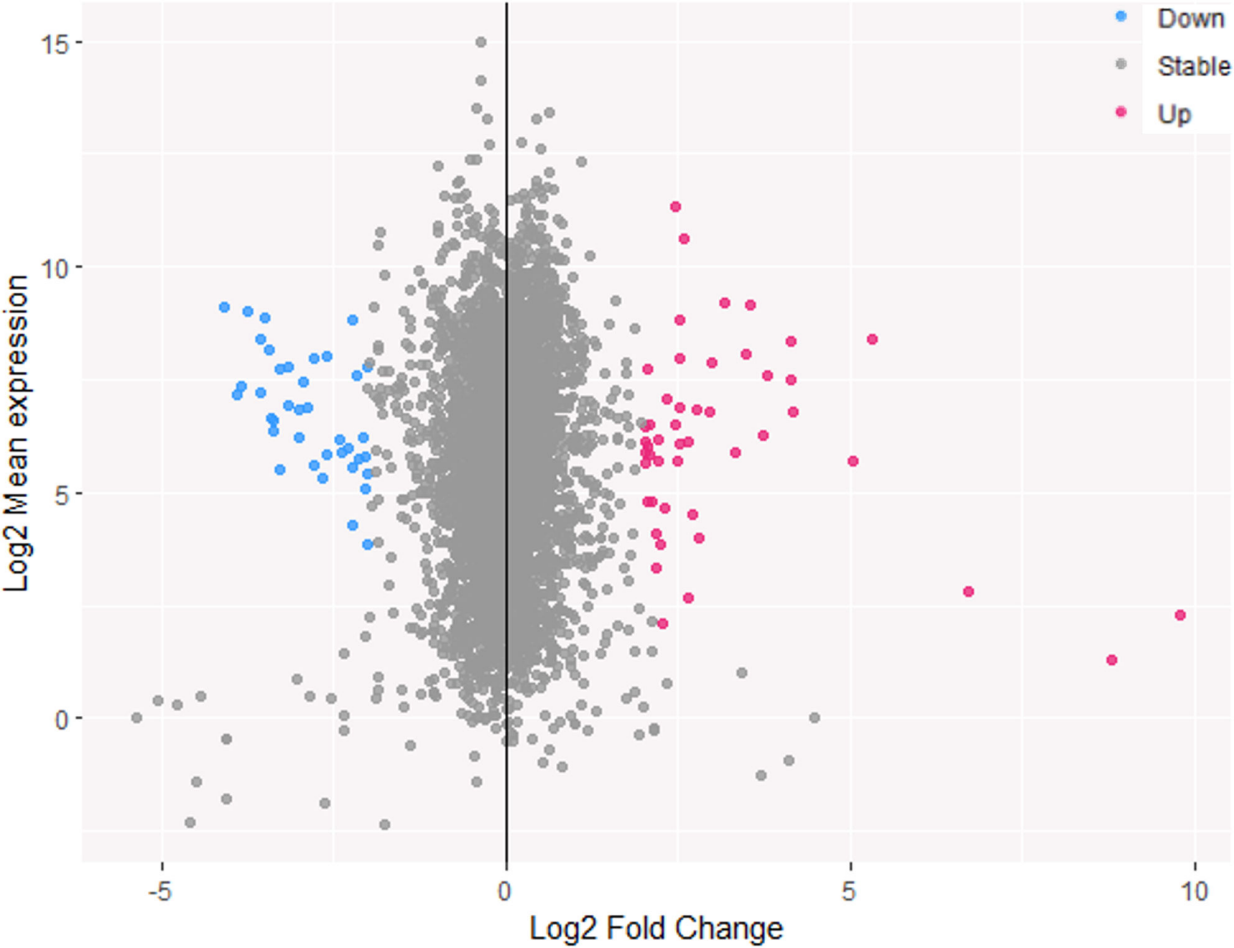
MA plot of the RNA results for *mmar_0009* mutant analysis. MA plot showing the Log2 mean expression and Log2 fold change of the differentially expressed genes. Upregulated genes (Log2 FC >2 and false discovery rate <0.005) are displayed in red and downregulated in blue (Log2 FC <2 and false discovery rate <0.005), gray for nondifferentially expressed genes.

Principal component analysis (PCA) revealed that the data of the two strains *cobC-metH* and *cobC-metH-mmar_0009* were separated; however, it is evident that the replicates’ clustering is mainly driven by PCA1 (Fig. S8). Quantitative analysis showed many differentially expressed genes. To ensure a robust analysis, we studied genes with a Log2 fold change (FC) ≥2 and ≤−2 and a false discovery rate of less than 0.05. From the total number of differential genes, 24 were downregulated, and 22 were upregulated, as displayed in the MA plot, representing Log2 FC versus Log2 mean expression, listed in Tables S2 and S3 (Fig. 6).

Interestingly, 83.33% of the downregulated genes had no direct homolog in Mtb. Mmar_0009 is not present in Mtb; therefore, it is probable that Mmar_0009 regulates specific genes for *M. marinum* (Fig. S9). We were interested in genes that could play a role in mycobacterial vitamin B_12_ scavenging. Therefore, since the tested mutant could now grow in the presence of B_12_, we expected that potential transporters would be downregulated (Table S2).

The gene *mmar_2973,* encoding for PE-PGRS62 (Rv3812), was significantly lower expressed (Log2 FC −2.61). The predicted structure of PE-PGRS62 (Alphafold) shows several α-helixes and four beta-sheets colored based on the per-residue confidence metric called predicted local distance difference test (pLDDT) (Fig. S10). The Mtb homolog displays more β-sheets forming a complete barrel. The pLDDT of the first 300 amino acids is overall lower than 70, whereas the region with the β-sheets is better predicted, with pLDDT higher than 70. This protein is annotated as a PE-PGRS protein; thus, we modeled the corresponding PE domain region, which presented a confident structure of the typical two-helix bundle (0–100 amino acids, 1.03 RMSD with PE25 from Mtb, PBD 4W4K, data not shown); however, this PE protein does not contain the characteristic glycine repeats observed in other PE-PGRS proteins (34).

Among the candidate list, one gene also had intriguing characteristics that could be linked to B_12_ scavenging. The gene *mmar_2554* encodes for a heme-binding protein based on structural homology studies (Rv0203 structural homolog, 64% coverage, 36% identity, RMSD 1.355, Log2 FC, −3.3). The Rv0203 is known to participate in the transport heme across the periplasm in Mtb (35). Molecules that share the same characteristics could be transported via the same transport system. Heme and vitamin B_12_ share the same core structure, the uroporphyrin ring (36). Thus, the B_12_ transport could also be mediated by a heme-binding protein.

Several genes involved in lipid biosynthesis were downregulated, fadD11_1 (Log2 FC −3.36), MMAR_1519 (Rv3130c, Tgs1, diacylglycerol O-acyltransferase, Log2 FC −3.01), and MMAR_3406 (encoding for a diacylglycerol o-acyltransferase, 98% confidence, 27% structural identity, Log2 FC −3.56). Again, membrane lipid composition seems to affect the transport of B_12_.

Among the highest-regulated genes, we found two genes belonging to the cysteine biosynthesis pathway and also directly linked to methionine synthesis (Table S3; Fig. S9). We also found ferredoxins to be differentially expressed in the *cobC-metH-mmar_009* mutant. Ferredoxins are iron–sulfur molecules that function as electron carriers (37, 38). We identified ferredoxins that were up- or downregulated. The *fdxA_2* gene was downregulated (Log 2 FC −3.43), whereas the *fdxA_1* and *fdxC_1* genes were upregulated (Log2 FC 2.51, 5.32).

## DISCUSSION

One of the most characteristic features of mycobacteria is their impermeable cell envelope that protects the bacterium. Simultaneously, the cell envelope must be specifically functionalized by proteins to allow the uptake of nutrients. Whereas the inner membrane resembles a barrier similar in composition and substrate transport systems to other diderm bacteria, little is known about how specific transport is achieved across the mycobacterial outer membrane (39, 40). Vitamin B_12_ is a large water-soluble molecule scavenged by mycobacteria from the growth medium (2, 12). While the transport mechanism of B_12_ across the inner membrane is well established, the underlying transport mechanism across the outer membrane remains unknown. In this work, we have shown that *M. marinum* can use exogenous B_12_ *in vitro* and *in vivo* and that alterations in the lipid membrane composition can affect this process. However, we were unable to identify a specific B_12_ outer membrane transporter.

The *de novo* synthesis pathway of methionine relies in the final step on a methyl-transferase reaction catalyzed by the two redundant enzymes MetH and MetE (6, 7, 9). MetH is a methionine synthase that requires B_12_ as a cofactor, whereas MetE is negatively regulated via a B_12_-sensitive riboswitch. Warner et al. (7) showed that in Mtb, individual mutants of each gene result in either a B_12_-auxotrophic (Δ*metE*) or B_12_-sensitive phenotype (Δ*metH*). By exploiting this regulated system, we set up a screen to identify new factors that play a role in the uptake mechanism of B_12_. It has not been conclusively determined through direct detection methods, such as mass spectrometry, whether *M. marinum* can produce B_12_. However, data from our group have verified active B12 biosynthesis (15). We required a strain that relied exclusively on exogenous B_12_ for our assays. Therefore, we constructed a mutant strain lacking the *cobC* gene belonging to the B_12_ biosynthesis. As expected, mutating the gene *metE* or the gene *metH* in the *cobC*::fs background gave the desired phenotypes, making these strains ideal for studying B_12_ uptake.

In contrast to most obligatory pathogens, *M. tuberculosis* is a prototroph organism with the biosynthetic capacity to produce amino acids, vitamins, and other cofactors from inorganic compounds without adding supplements (41). Recent findings suggest that Mtb cannot produce vitamin B_12_ anymore as one of the *cob* genes is corrupted, *cobF* (6). This would mean that Mtb depends on exogenous B_12_ from the host since the B_12_-dependent enzymes are still produced and functional. We employed the zebrafish embryo infection model to investigate whether *M. marinum* can access and transport B_12_ *in vivo*. When infecting the embryos with the *cobC* mutant of *M. marinum*, the mutant was moderately but significantly less virulent compared to the WT strain. This could indicate that *M. marinum* indeed produces vitamin B_12_ during infection and that this host-independent reservoir of B_12_ is advantageous for growth within the host. Because the *cobC-metE* mutant showed the same moderate virulence defect as the *cobC* mutant, it can be concluded that B_12_ is present during infection, and *M. marinum* can sufficiently scavenge it to fulfill its metabolic needs. The reciprocal mutation, i.e., c*obC-metH* mutant, was strongly attenuated.

To elucidate the transport mechanism of B_12_, we used two complementary approaches. Our first approach using the *nucS-cobC-metE* mutant was aimed to identify mutations that could improve B_12_ transport. Whole-genome sequencing of a selected mutant revealed three mutations in a PKS operon (*mmar_3097-mmar_3098*). In addition, a frameshift mutation in the gene *ppsC*, *mmar_1774*, was identified. The low permeability of mycobacteria impacts bacterial survival inside the host macrophage as antimicrobial compounds cannot penetrate the cell envelope (40). The mycobacterial growth rate is lower than other known pathogens due to reduced nutrient uptake (12, 40). Therefore, increased membrane permeability could result in improved transport of bulky molecules such as vitamin B_12_. Unfortunately, obvious candidates of proteins involved in active outer membrane transporter were not identified.

Our second approach made use of a saturated transposon library in the *cobC-metH* mutant background to select, in this context, transposon mutants resistant to B_12_. Selected mutants grew in a 10-fold higher concentration of B_12_ than the parental *cobC-metH* mutant strain. Two transposon insertions in eight individual mutants were found in *mmar_3798*, a PKS-1 enzyme involved in lipid metabolism and cell wall assembly, and one transposon hit was found in the gene *mmar_0407*, a potential lipid transporter also involved in lipid metabolism.

Additionally, transposon mutants of the *esxU-esxT* promoter and gene region resulted in a B_12_-resistant phenotype in the *cobC-metH* mutant. These two genes encode for two substrates of the ESX-4 Type VII secretion system (42). One striking difference between the B_12_ uptake of mycobacteria and gram-negative bacteria is the efficiency. Mycobacteria require 10–20 times more extracellular B_12_ than gram-negative bacteria, questioning the existence of a high-affinity uptake system in the mycobacterial outer membrane (43, 44). Contrary to this, we collected evidence that *M. marinum* gains access to B_12_ *in vivo* during infection. The concentration of B_12_ in zebrafish must certainly be lower than the minimum concentration we can use *in vitro* experiments (100 ng/mL) to stimulate the growth of our *cobC* mutants, suggesting that uptake during infection is more efficient *in vivo* (45). It is possible that infection serves as a signal to express such a high-affinity uptake system, which would not be needed under culture conditions, although one would expect that when selecting in our screens for induced expression of such a system. In this study, we identified EsxUT, a vital component to secrete protein substrates of the ESX-4 secretion system. ESX-4 is known to be inactive under regular culture conditions but likely expressed under certain conditions such as infection (46, 47). We hypothesized that an ESX-4 substrate could facilitate B_12_ uptake across the outer membrane during infection. Except for one knockdown mutant (KDmmar_0407, a predicted lipid transporter), all potential gene candidates in *M. marinum* showed reduced dye uptake compared to WT, suggesting a general increase in cell wall permeability rather than a specific lack of B_12_ uptake, indicating again that membrane integrity strongly affects the transport of B_12_ in mycobacteria. Interestingly, a prominent gene on our list of B_12_-resistant transposon mutants is the gene *mmar_0009*, identified from two independent insertions in three selected mutants. Protein homology studies allow us to identify this gene as a potential MerR stress response transcription regulator (48). The two transposon locations were found in the sensor domain of Mmar_0009, suggesting that blocking the sensor binding of Mmar_0009 could allow growth on B_12_ in the *cobC-metH* mutant context. The predicted structure of the C-terminal sensor domain was close to the TipAS domain of the TipAL protein in *Streptomyces lividans*. Thiopeptides are formed by a macrocyclic core ring (32). This ring structure is comparable to the porphyrin ring found in vitamin B_12_. Strikingly, in 2003, Kahmann et al. identified the basis of the recognition site of the TipA molecule and discovered that this binding was similar to the classical globin binding that can accommodate heme and other tetrapyrroles (33). We speculated that the sensor domain might bind to B_12_ and regulate downstream genes involved in B_12_ uptake. We performed global transcriptomics analyses to elucidate the regulon of Mmar_0009 and shed light on the B_12_-resistant phenotype.

Many genes appeared to be affected by the absence of Mmar_0009. Again, genes related to lipid metabolism were highly affected in the *cobC-metH-mmar_0009* mutant. Throughout our study, we have seen with our different assays how membrane integrity impacts the uptake of the water-soluble molecule vitamin B_12_. Hydrophilic compounds cannot traverse the extremely hydrophobic mycomembrane. Considering all the data, it could be possible that the outer membrane, as a dynamic layer of the cell, changes its permeability deliberately to allow the passage of important hydrophilic and bulky molecules, even if this entails local or partial vulnerability of its protective properties. Based on our results, we could speculate that Mmar_0009 senses vitamin B_12_ inside the bacterium and controls the expression of these membrane lipid synthases to restore the integrity of the membrane.

We also highlighted the gene *mmar_2973,* encoding for PE-PGRS62 (Rv3812), that was downregulated in the mutant *cobC-metH-mmar_0009* when compared to its parental *cobC-metH* mutant strain. This protein is annotated as a PE-PGRS protein (PE-PGRS62), although neither the sequence of the protein nor its *M. tuberculosis* ortholog contains the characteristic glycine-rich repeats or the GRPLI domain directly after the PE domain (34). The predicted structure of MMAR_2973 presents two α-helixes in the N-terminal domain followed by the YxxxD/E motif, characteristic of a type VII secretion protein substrate. The C-terminal domain is predicted to form another set of alpha-helices and β-sheets that form an unusual β-barrel-like structure perpendicular to the α-helixes. This β-barrel is more evident in the homolog protein Rv3812, which forms a complete closed conformation. This structure should be investigated in more detail to see whether it can function as a pore allocating the bulky vitamin B_12_. Mmar_2554, one predicted secreted protein, was also highly downregulated (Log2 FC −3.3). Structural analysis showed that Mmar_2554 is close to the heme-binding protein Rv0203 (RMSD 1.355) involved in the transport of heme in Mtb *in vitro* (35). Heme shares structural properties with vitamin B_12_ (35, 36). Mmar_2554 could, therefore, possibly bind the corrin ring of vitamin B_12_, similar to the porphyrin ring in heme having the function of a transporter. However, it is important to exercise caution with conclusions as these results are derived from a global transcriptomic study, and therefore, we only acknowledge their potential involvement in the transport process. Moreover, among all the genes we have found throughout our study, many have no homolog in Mtb. As previously mentioned, Mtb cannot synthesize B_12_ and, therefore, does not need to switch between scavenging or synthesizing B_12_. Indeed, the *mmar_0009* regulator has no direct homolog in Mtb; thus, it could be that its regulon is *M. marinum*-specific.

Intriguing in our results is the absence of the inner transporter (Rv1819c, MMAR_2696), probably due to some limitations in our assays. Thus, with the data we obtained, we cannot exclude that there is an active transport channel in the outer membrane.

In conclusion, we have shown that alterations in the membrane integrity and permeability strongly affect the uptake of B_12_ across the cell envelope. This highlights the importance of the membrane’s impermeable status, and when alterations happen, big molecules can get through it without needing any transport system. Currently, only the inner-membrane transporter of vitamin B_12_ has been elucidated (Rv1819c). Understanding the uptake of big molecules such as vitamin B_12_ could help innovate and investigate new delivery methods for potential antimicrobials to fight tuberculosis disease.

## MATERIALS AND METHODS

### Bacterial strains and cell cultures

*Mycobacterium marinum* M^USA^ was used as the parental strain for all *M. marinum-*derived strains (Table S4). The mycobacterial strains were routinely grown at 30°C in liquid Middlebrook (Difco-BD Biosciences) 7H9 or solid Middlebrook 7H10 medium supplemented with 10% (vol/vol) ADS [5% (wt/vol) albumin, 2% (wt/vol) dextrose, and 0.16% (wt/vol) NaCl], 0.2% glycerol, and 0.01% (vol/vol) tyloxapol. *Escherichia coli* Dh5α, used to construct and propagate plasmids, was grown in Luria–Bertani liquid and solid medium at 37°C. Where necessary, the antibiotics kanamycin (Kan, 25 µg/mL) and hygromycin (Hyg, 50 µg/mL) were added to the medium.

### Construction of frameshift and knockdown mutants

All strains and plasmids used within this study are described in Tables S4 and S5, respectively. The oligonucleotides encoding sgRNAs used for the frameshift mutants are listed in Table S6. Guide RNAs were ordered as oligonucleotides and hybridized. The resulting dsDNA oligonucleotides were ligated into the backbone pCRISPRx-Sth1Cas9-L5, which was opened with the restriction enzyme BsmBI. The expression of a specific sgRNA allowed CRISPR/Cas9 to cut the chromosomal DNA at the targeted gene. The innate DNA repair mechanisms of the bacterial cell (nonhomologous end joining) introduced or removed nucleotides during the repair of the double DNA break. In the selected cases, this led to the creation of frameshift mutations in the targeted genes. The target site prediction and the generation of mutants were performed as previously described (49). Briefly, CRISPR/Cas9 plasmids containing a sgRNA targeting the gene of interest were transformed into *M. marinum* and selected on a solid medium containing 200 ng/mL of anhydrotetracycline (ATc) to induce the expression of *cas9*. Selected bacterial colonies were isolated, and the Cas9-targeted genomic region was amplified by PCR and sequenced to identify frameshift mutants. Mutations generated to create the frameshift mutants used in this study are shown in Fig. S11. Sequential mutants could be created by swapping the CrisprCas9 plasmid (Kana^R^), an identical vector with exchanged resistance cassette (Hyg^R^). In a similar fashion, we constructed gene knockdown mutants of selected genes. The oligonucleotides encoding the sgRNAs listed in Table S4 were cloned in an identical procedure into pLJR962. This backbone contains ATc inducible Cas9 with a mutated catalytical domain, preventing DNA cleavage. Upon expression, the sgRNA-loaded Cas9 enzyme binds specifically to the targeted gene and prevents RNA polymerase from transcribing the gene, resulting in a gene knockdown.

### Construction of gene deletion mutant

The *M. marinum nucS* (*mmar_4077*) deletion strain was generated by homolog recombination using specialized transduction for the introduction of the deletion construct into the bacterium, following the method published by Bardarov et al. (50). The flanking regions of *nucS* were amplified with primer pairs NucS_KO_LF/NucS_KO_LR and NucS_KO_RF/NucS_KO_RR (Table S7), resulting in a 1,086- and 1,183-bp-long amplicon, respectively. After the selection for a recombination event, candidates were verified by PCR analysis and sequencing. About 93% of the original *nucS* gene was deleted from the genome. We employed a temperature-sensitive phage harboring the γδresolvase (*tnpR*) gene to excise the resistance genes, resulting in an unmarked deletion mutation.

### Construction of expression vectors

Expression plasmids pSMT3-mmar0009 were constructed by amplifying *mmar_0009* from *M. marinum* chromosomal DNA using primer pairs Mmar0009-FW/Mmar0009-RV (Table S7). The resulting amplicon was digested with the restriction enzymes NheI and SpeI and then ligated into the backbone pSMT3, which had been previously digested with the same enzymes. Plasmid pSMT3-metH was created by initially amplifying the gene *metH* (*rv2124c*) from the chromosomal DNA of Mtb H37Rv using primer pairs MetH-FW/MetH-RV (Table S7). The resulting amplicon was then digested with PacI and ClaI before being ligated into the backbone pMN016, which had been opened using the same enzymes.

### Growth assay on B_12_ medium

Bacteria were grown in 7H9-ADS-glycerol-tyloxapol liquid medium supplemented with appropriate antibiotics until they reached the mid-logarithmic phase, with OD_600_ of 0.8–1.2. Bacteria were then washed with phosphate-buffered saline (PBS) and diluted to an OD_600_ of 0.1. For the solid growth assays, serial 10-fold dilutions were made, and bacteria were plated on 7H10 plates containing different concentrations of vitamin B_12_. For liquid growth assays, growth was monitored every 12–24 h until they reached the stationary phase. Selective media were the 7H9 medium containing 1 µg/mL B_12_, 1 mM methionine, and a combination of both.

### Zebrafish infection studies

Transparent zebrafish embryos (Casper) were infected at 1 day post-fertilization via the caudal vein with a bacterial suspension containing 50–200 CFU. The injection was performed as described previously (51). The injection volume was plated on 7H10 plates to determine the exact number of injected bacteria. At 5 days post-infection, embryos were analyzed with an Olympus IX83 fluorescence microscope. Infection levels were quantified by counting pixels of the fluorescent signal using an automated image analysis software (CellProfiler) as explained previously (52).

### Selection on B_12_ gradient plates and characterization of resulting mutants

The Δ*nucS cobC*::fs-*metE*::fs mutant was grown in the 7H9-ADS-glycerol-tyloxapol medium supplemented with appropriate antibiotics until the mid-logarithmic phase (OD_600_ 0.8–1.2). After being washed with PBS, 2 × 10^5^ CFU/plate of bacteria was plated on a solid medium containing a B_12_ concentration gradient. Gradient plates were prepared as illustrated in Fig. 4. Briefly, the lower agar layer containing the medium supplemented with 100 ng/mL of B_12_ was poured into a tilted plate and solidified. Then, the plate was moved to a leveled surface, and the upper layer with the 7H10 medium was poured, creating a homogeneous gradient of different B_12_ concentrations.

### Whole-genome sequencing

The chromosomal DNA of potential mutant candidates was isolated using the acid guanidinium thiocyanate–phenol–chloroform extraction method. Washed in 80% ethanol and resuspended in 10 mM Tris, the integrity of the DNA and absence of RNA were verified by gel electrophoresis. The DNA was subjected to Illumina sequencing (Novogene Bioinformatics Technology). Raw pair-end reads were analyzed with CLC Genomics Workbench 12. Reads were quality-filtered and trimmed using Illumina pipeline 1.8 based on the Phred score (limit score 0.05). Illumina adapters used for the sequencing procedure were trimmed and mapped to the reference genome *M. marinum* M^USA^ (NC_010612.1) using a mapping score of 1, mismatch cost of 2 and gap insertion/deletion cost of 3, length fraction of 0.5 and similarity fraction of 0.8, and local alignment and selecting the nonspecific match random handling setting. Mapped reads were then analyzed for variations including SNPs (single-nucleotide polymorphism) and insertions and deletions with a minimum of two reads showing a mismatch.

### Transposon mutagenesis

A transposon library was constructed in the *cobC::fs-metH::fs* mutant strain. Bacteria were infected with the mycobacterial phage phiMycoMarT7 containing the *himar1* transposon with a kanamycin resistance cassette, as described (53). 2 × 10^4^ CFU/mL bacteria were plated on a selective medium containing high B_12_ levels (1 µg/mL). Screening experiments were performed three times using two plates per condition. B_12_-resistant colonies were isolated to determine the transposon insertion site by ligation-mediated PCR (54).

### EtBr uptake experiments

Cells were grown in a 7H9-ADS-glycerol-tyloxapol medium supplemented with appropriate antibiotics until they reached the mid-logarithmic phase at OD_600_ 0.8–1.2. On the day of the experiment, bacteria were washed twice with PBS and normalized in PBS to a final OD_600_ of 1. EtBr was added to the bacterial suspension to a final concentration of 10 µg/mL. Fluorescence was measured every 3 minutes for 3–6 h (excitation: 300 nm/emission: 605 nm) using a plate reader.

### Extraction, sequencing, and analysis of mRNA

RNA isolation was performed by following the recommendations of a NucleoSpin RNA kit (Macherey-Nagel). In total, the RNA of 25 OD units per biological replicate was isolated. Cells were disrupted by 0.1-mm zirconium beads in 500 µL buffer RA1 and 5 µL β-mercaptoethanol for 1 minute. Subsequent steps were performed according to the protocol provided by the manufacturer. RNA concentrations were determined by spectroscopy. The RNA was considered pure when the absorbance 260/280 nm ratio was above 2. For quality control, RNA samples were electrophoretically separated on a 1% agarose gel to control for RNA integrity and contamination with chromosomal DNA. The RNA sequencing was performed by Illumina sequencing (Novogene Bioinformatics Technology). The RiboMinus prokaryotic kit (Invitrogen) was employed to deplete rRNA and enrich mRNA. RNA sequencing analysis was performed by outsourcing the mRNA to Novogene. The obtained readouts were analyzed using CLC Genomics Workbench 12, toolbox RNA-Seq Analysis. The reads underwent a screening process for quality using Illumina pipeline 1.8 based on the Phred score (limit score 0.05). Illumina adapters were also trimmed. Trimmed reads were then mapped to the *M. marinum* M^USA^ reference genome (NC_010612.1) containing the annotated genes and using mapping scores of 1, mismatch cost of 2 and gap insertion/deletion cost of 3, length fraction of 0.5 and similarity fraction of 0.8, local alignment, and nonspecific match random handling a maximum number of hits for a read of 10. Count data were then converted to reads per million mapped reads and subjected to differential analysis. Gene function and classification were ascertained by utilizing Mycobrowser (https://mycobrowser.epfl.ch/) or, in the absence of the aforementioned, the protein fold recognition homology search engine phyre2 (http://www.sbg.bio.ic.ac.uk/phyre2/html/page.cgi?id=index).

## Data Availability

Whole-genome sequencing data have been deposited to the Sequence Read Archive (SRA) within the Accession Bioproject PRJNA1003652. RNA sequencing data have been deposited to the Gene Expression Omnibus (GEO) under accession number GSE240448.

## SUPPLEMENTAL MATERIAL

Supplemental legends spectrum.03168-23-s0001.docx Legends describing the supplemental figures and tables.

Supplemental material spectrum.03168-23-s0002.docx Fig. S1-S11; Tables S1-S7.

ASM does not own the copyrights to Supplemental Material that may be linked to, or accessed through, an article. The authors have granted ASM a non-exclusive, world-wide license to publish the Supplemental Material files. Please contact the corresponding author directly for reuse.
