# Supplementary material for: Vitamin B12 uptake across the mycobacterial outer membrane is influenced by membrane permeability in Mycobacterium marinum
Source: Microbiol Spectr. 2024 May 9;12(6):e03168-23. doi: 10.1128/spectrum.03168-23 (PMC11237697; doi:10.1128/spectrum.03168-23)
Supplement: Supplemental legends — Legends describing the supplemental figures and tables. [file spectrum.03168-23-s0001.docx]

**SUPPLEMENTARY FIGURE LEGENDS**

**SUPPLEMENTARY FIGURE 1. *CobC*::fs-*metE*::fs chemical complementation*.*** *CobC*::fs-*metE*::fs mutant at OD_600_ of 0.05 were grown first with 1 µg/mL of B_12_ and monitored for growth every 24h by OD_600_ measurements. When the logarithmic phase was reached (OD_600_ around 1), bacteria were diluted back to OD_600_ 0.05 and grown in media without B_12_ and with or without 0.5 mM methionine.

**SUPPLEMENTARY FIGURE 2. *CobC*::fs-*metH*::fs chemical complementation*.*** The *cobC*::fs-*metH*::fs mutant was inoculated at OD_600_ 0.05 and incubated with and without 1 µg/mL of B_12_ and with and without 0,5 mM methionine. Growth was monitored every 24h by OD_600_ measurements.

**SUPPLEMENTARY FIGURE 3. *CobC*::fs-*metH*::fs and *CobC*::fs-*metH*::fs-*mmar_0009*::fs genetical complementation*.***  Mutant were plated at OD_600_ 0.1 and incubated with and without 1 µg/mL of B_12_ until colonies were visible.

**SUPPLEMENTARY FIGURE 4.** EtBr uptake experiment to determine membrane permeability. Bacteria were exposed to 10 µg/mL of EtBr and fluorescence was measured every 3 minutes (ex:300 nm/em:605 nm). WT was used as a reference while WT expressing *mspA,* the mutant strains lacking *ppsE*::fs or *papA5*::fs, served as representative strains with increased membrane permeability.

**SUPPLEMENTARY FIGURE 5**. Ethidium bromide (EtBr) uptake assay to assess membrane permeability in additional mutants selected from the Δ*nucS* *cobC:*:fs-*metE*::fs background. Bacteria were treated with 10 µg/mL of EtBr, with fluorescence intensity recorded every 3 minutes (ex: 300 nm/em: 605 nm). A Δ*nucS* mutant served as a reference for normal EtBr uptake.

**SUPPLEMENTARY FIGURE 6.** Ethidium bromide (EtBr) uptake assay to evaluate membrane permeability in knock-down mutants *M. marinum* WT background identified via transposon mutagenesis. Bacteria were transformed with pLJR965 carrying specific sgRNAs and cultured to mid-logarithmic phase (OD 1.5-2.0) in the presence of 200 μg/mL ATc to induce gene knockdown. Following induction, bacteria were treated with 10 µg/mL of EtBr, fluorescence intensity measured every 3 minutes (ex: 300 nm/em: 605 nm). A wild-type (WT) mutant, also exposed to ATc, served as a reference. "KD" designates knock-down mutants.

**SUPPLEMENTARY FIGURE 7. The B_12_-resistant phenotype of two *cobC*::fs*-metH::*fs *–mmar_0009*::fs mutant strains.** Growth assays on solid media supplemented with 0, 100 and 1000 ng/mL B_12_ . Strains serving as additional controls are WT, *cobC*::fs, *cobC*::fs-*metH*::fs.

**SUPPLEMENTARY FIGURE 8. Principal component analysis (PCA) of the normalized RNA transcripts for *mmar_0009* mutant analysis.** Triplicates of technical replicates were analyzed to check for the variability of the data. The mutant strain *cobC*-*metH* is displayed in red (AmetH_1, BmetH_1, CmetH_1) and in blue, *cobC*-*metH*-*mmar_0009* mutant (A0009_1, B0009_1, C0009_1).

**SUPPLEMENTARY FIGURE 9. RNA sequencing results comparing *cobC*::fs-*metH*::fs to *cobC*::fs-*metH*::fs-*mmar_0009*::fs in *M. marinum*.** Sequenced RNA was trimmed and aligned to the reference genome of *M. marinum* M^USA^ (NC_010612.1). For differential gene expression, fold change ≥ 2 ≤, false discovery rate 0.05. Functional category plot with all the categories in the Y-axis and the X-axis Log 2-fold change in blue homologs in Mtb.

**SUPPLEMENTARY FIGURE 10. Alphafold protein structure prediction of Mmar_2973.** The predicted structure of Mmar_2973 and its homologue in Mtb Rv3812, named PE_PGRS62. The colors correspond to the per-residue confidence score (pLDDT). Dark blue (very high confidence) pLDDT > 90, light blue 90 > pLDDT > 70, yellow 70 > pLDDT > 50 and orange (very low confidence) <50.

**SUPPLEMENTARY FIGURE 11. Comprehensive illustration of frame shift mutants utilized in this investigation.** The guide RNA sequences (sgRNA) directing the Cas9 enzyme to specific genomic sites are highlighted in purple boxes. Genomic PAM sequences essential for Cas9 binding to the genomic region are denoted by grey boxes (PAM), situated two nucleotides away from the guide RNAs. The figure shows the wild-type (WT) gene and its translated product. Following Cas9-induced DNA cleavage, repair by the bacterial cell via non-homologous DNA end joining (NHEJ) results in the insertion or deletion of nucleotides, illustrated by highlighted DNA segments in pink. In the depicted instances, this process leads to the generation of frame shift mutations.

**SUPPLEMENTARY TABLE LEGENDS**

**SUPPLEMENTARY TABLE 1. Whole Genome sequencing results of *nucS*-*cobC*-*metE-*_parental_.** Chromosomal DNA extraction was performed on the parental *nucS*-*cobC*-*metE* mutant strain and further analyzed by the whole genome sequencing using a Illumina platform by Beijing Novogene Bioinformatics Technology (Novogene). Raw pair-end reads were analyzed with Qiagen CLC Genomics Workbench and aligned to the reference genome of *M. marinum* M^USA^ (NC_010612.1). Strains were mapped and checked for variations and SNPs. SNV: Single-nucleotide variants; MNV: Multi-nucleotide variants.

**SUPPLEMENTARY TABLE 2**. **Downregulated genes from the RNA sequencing *cobC*::fs-*metH*::fs vs *cobC*::fs-*metH*::fs-*mmar_0009*::fs**. List of downregulated genes with a Log2 FC≤-2 and false discovery rate <0.005. Genes are described by their functional category and Mtb homologs.

**SUPPLEMENTARY TABLE 3. Upregulated genes from the RNA sequencing *cobC*::fs-*metH*::fs vs *cobC*::fs-*metH*::fs-*mmar_0009*::fs**. List of upregulated genes with a Log2 FC≥2 and false discovery rate <0.005. Genes are described by their functional category and Mtb homologs.

**SUPPLEMENTARY TABLE 4**. **List of strains used in this study.** Strains used in this study with their specific characteristics and parental strains description.

**SUPPLEMENTARY TABLE 5. List of plasmids used in this study.** Plasmids used in this study with their specific characteristics.

**SUPPLEMENTARY TABLE 6. List of oligonucleotides encoding sgRNAs. Green** oligonucleotides were used to construct derivatives of pCRISPRx-Sth1Cas9-L5 targeting specific genomic locations ^51^. **Blue** oligonucleotides were used to construct derivatives of pLJR962 to construct knock-down mutants (**Fig. S6**) ^30^. The restriction enzyme BsmbI was used to introduce the oligo in the plasmids. Knock-down strength for each PAM sequence is indicated as fold repression, values have been determined by Rock *et al*., 2017 ^30^.

**SUPPLEMENTARY TABLE 7. List of oligonucleotides used to construct plasmids.**
