# Supplementary material for: Vitamin B12 uptake across the mycobacterial outer membrane is influenced by membrane permeability in Mycobacterium marinum
Source: Microbiol Spectr. 2024 May 9;12(6):e03168-23. doi: 10.1128/spectrum.03168-23 (PMC11237697; doi:10.1128/spectrum.03168-23)
Supplement: Supplemental material — Fig. S1-S11; Tables S1-S7. [file spectrum.03168-23-s0002.docx]

**SUPPLEMENTARY FIGURES**

**Fig.S1**


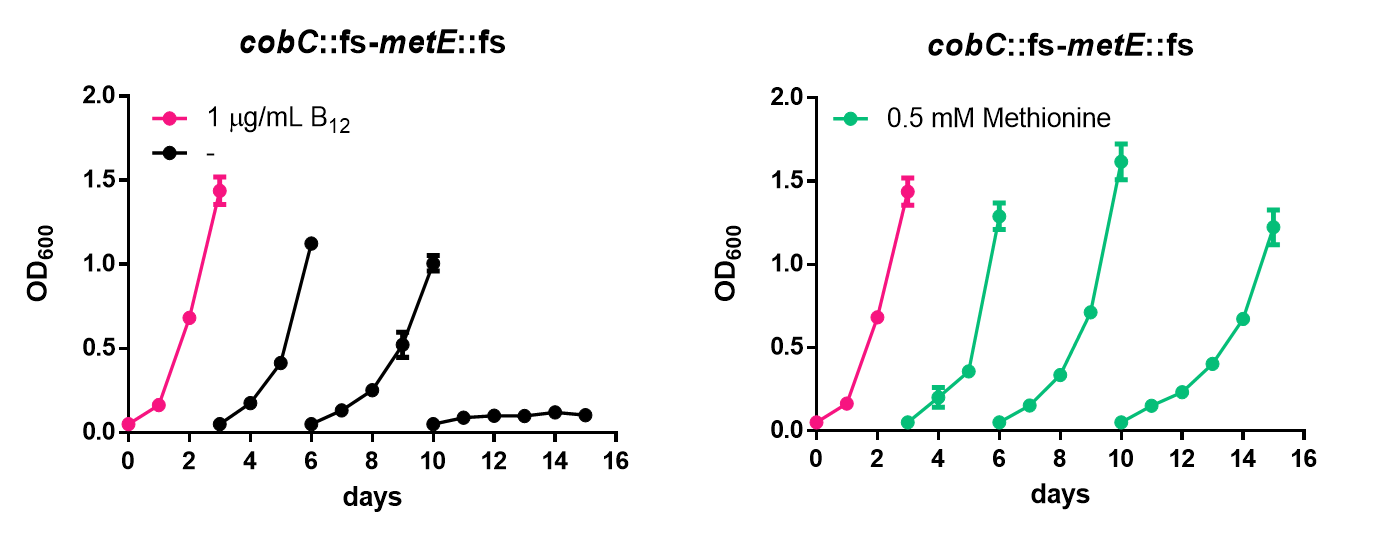


**Fig.S2**


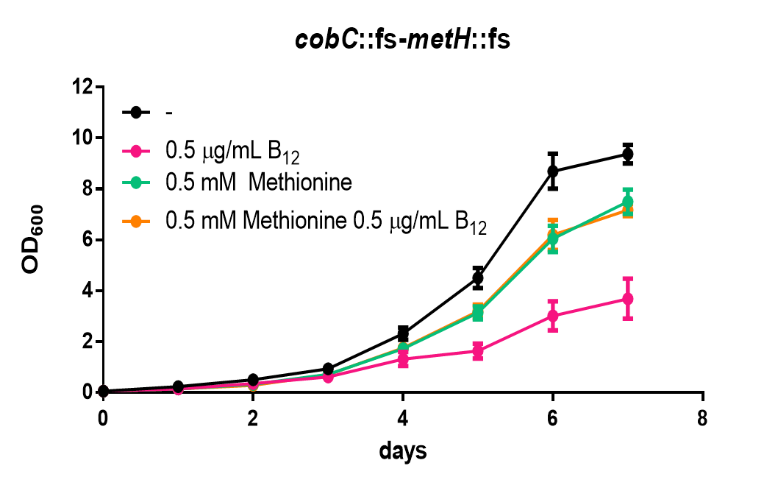


**Fig.S3**


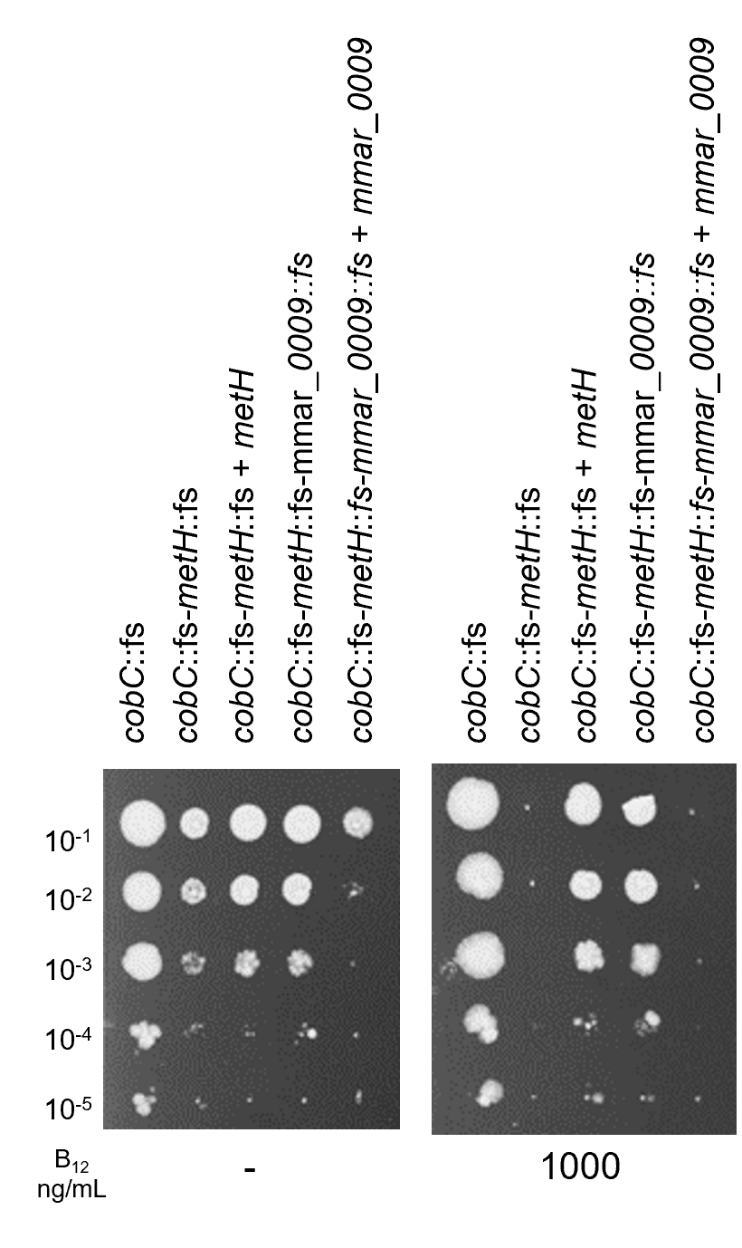


**Fig.S4**


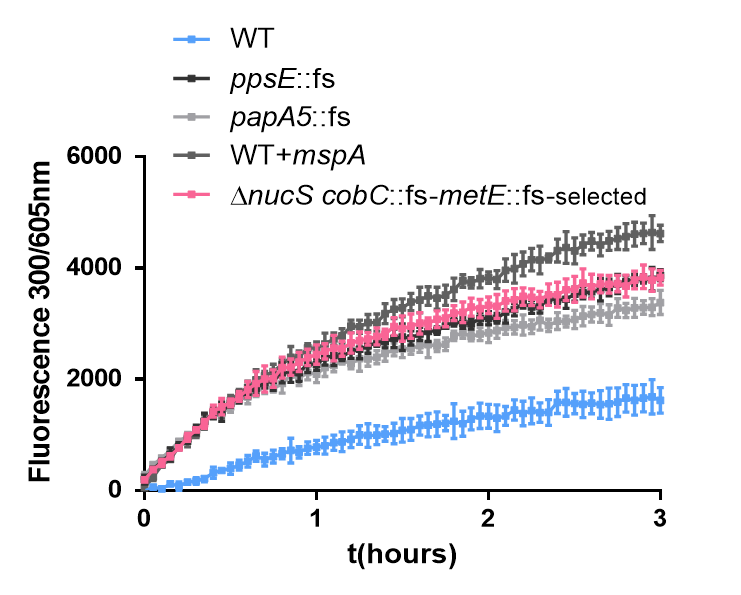


**Fig.S5**


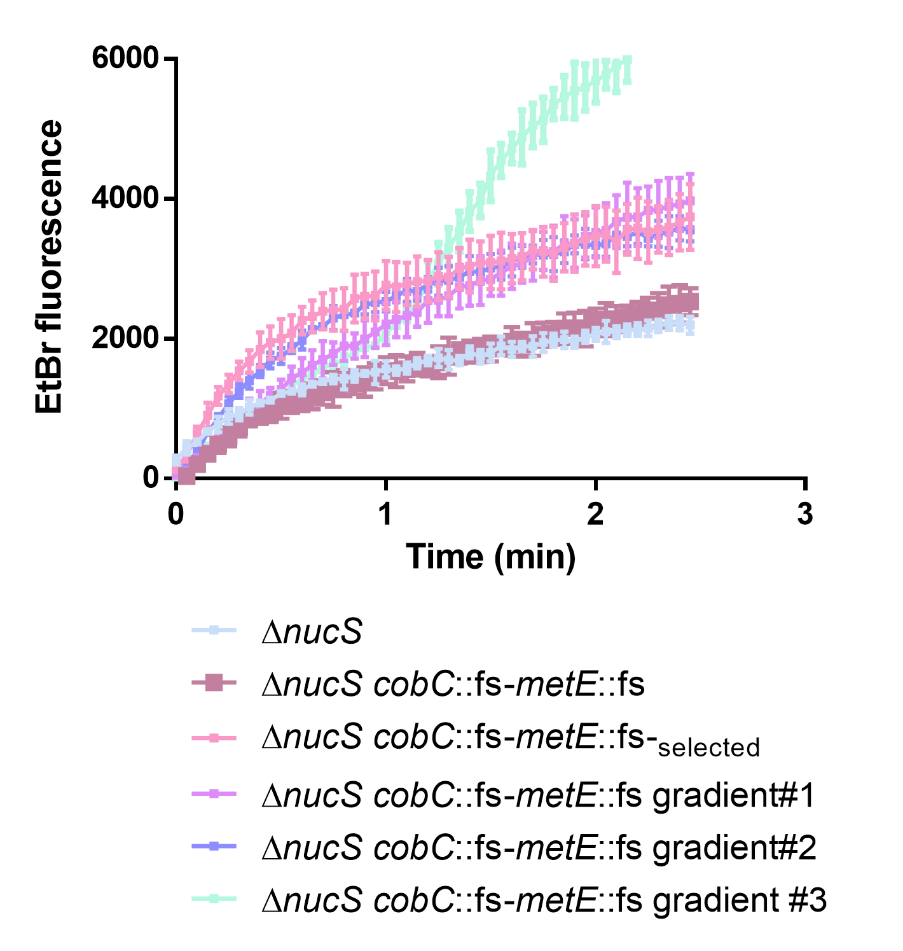


**Fig.S6**


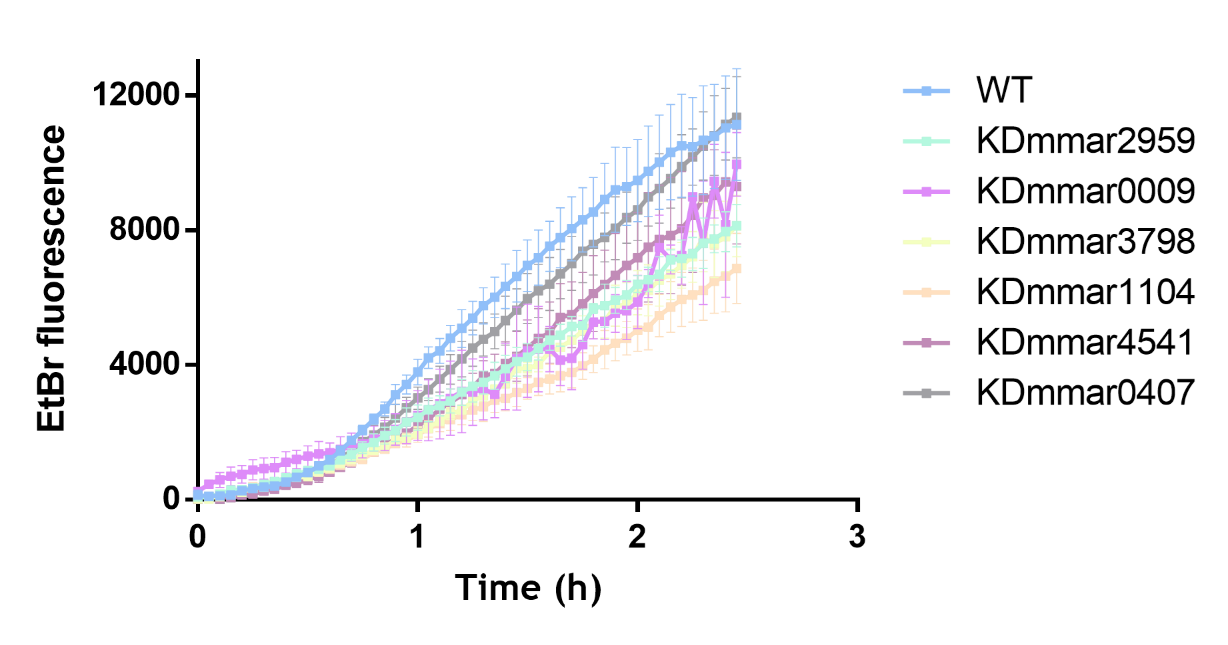


**Fig.S7**


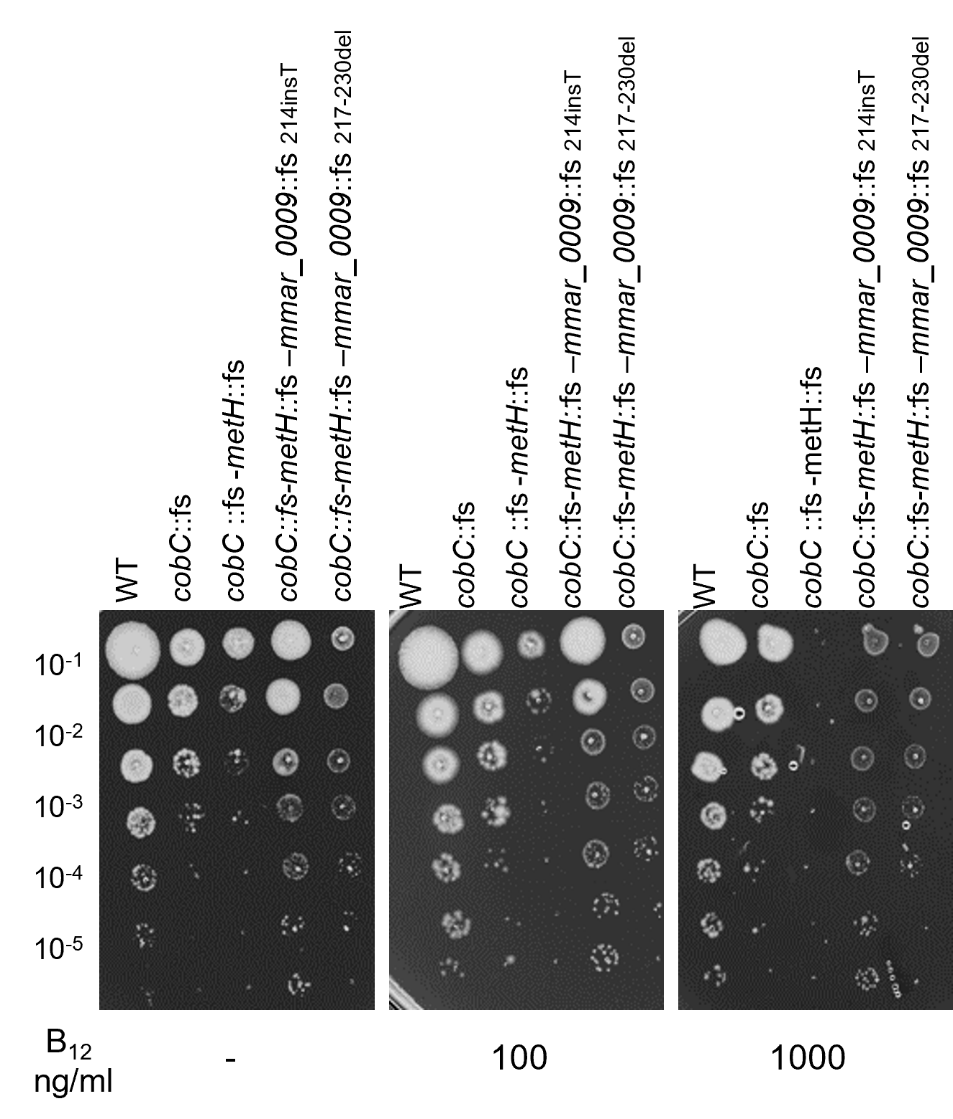


**Fig.S8**


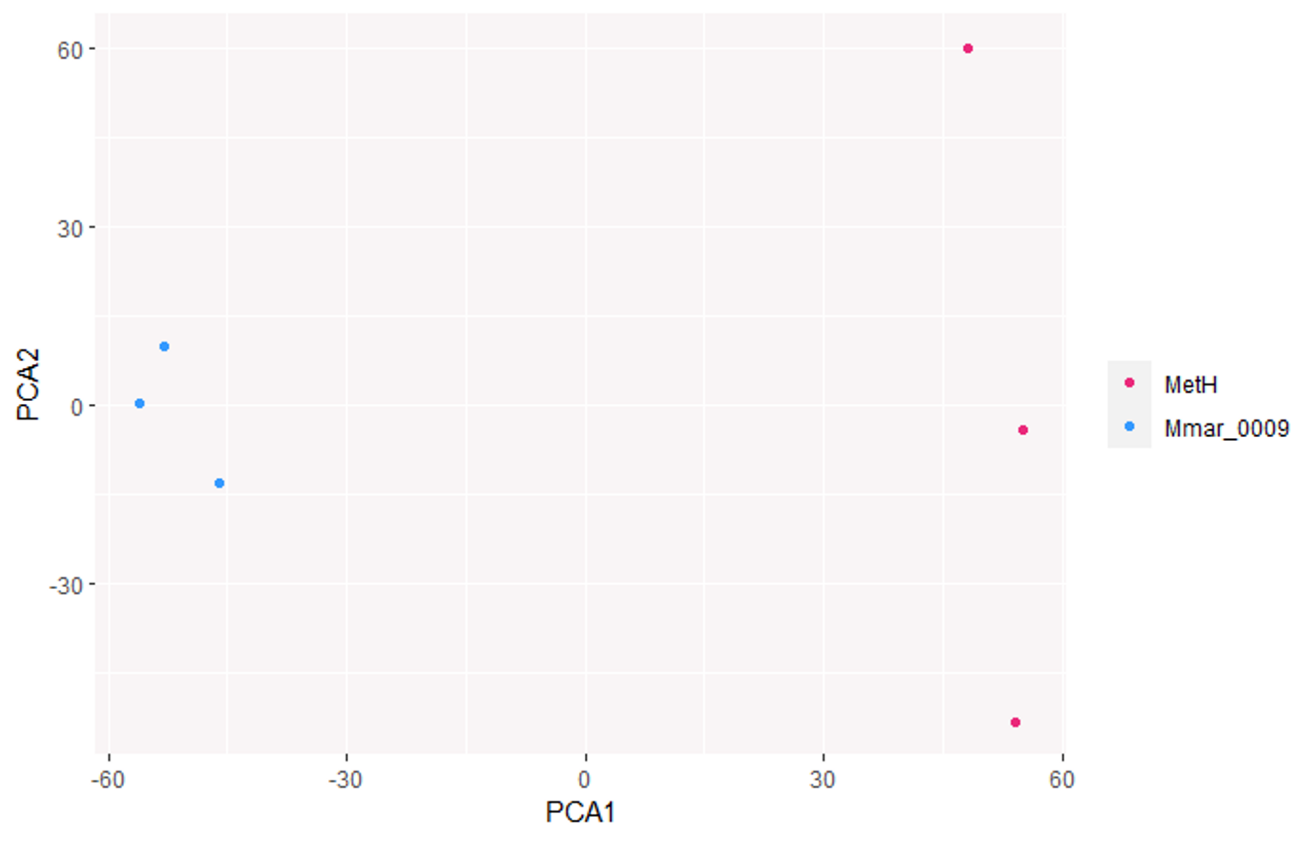


**Fig.S9**


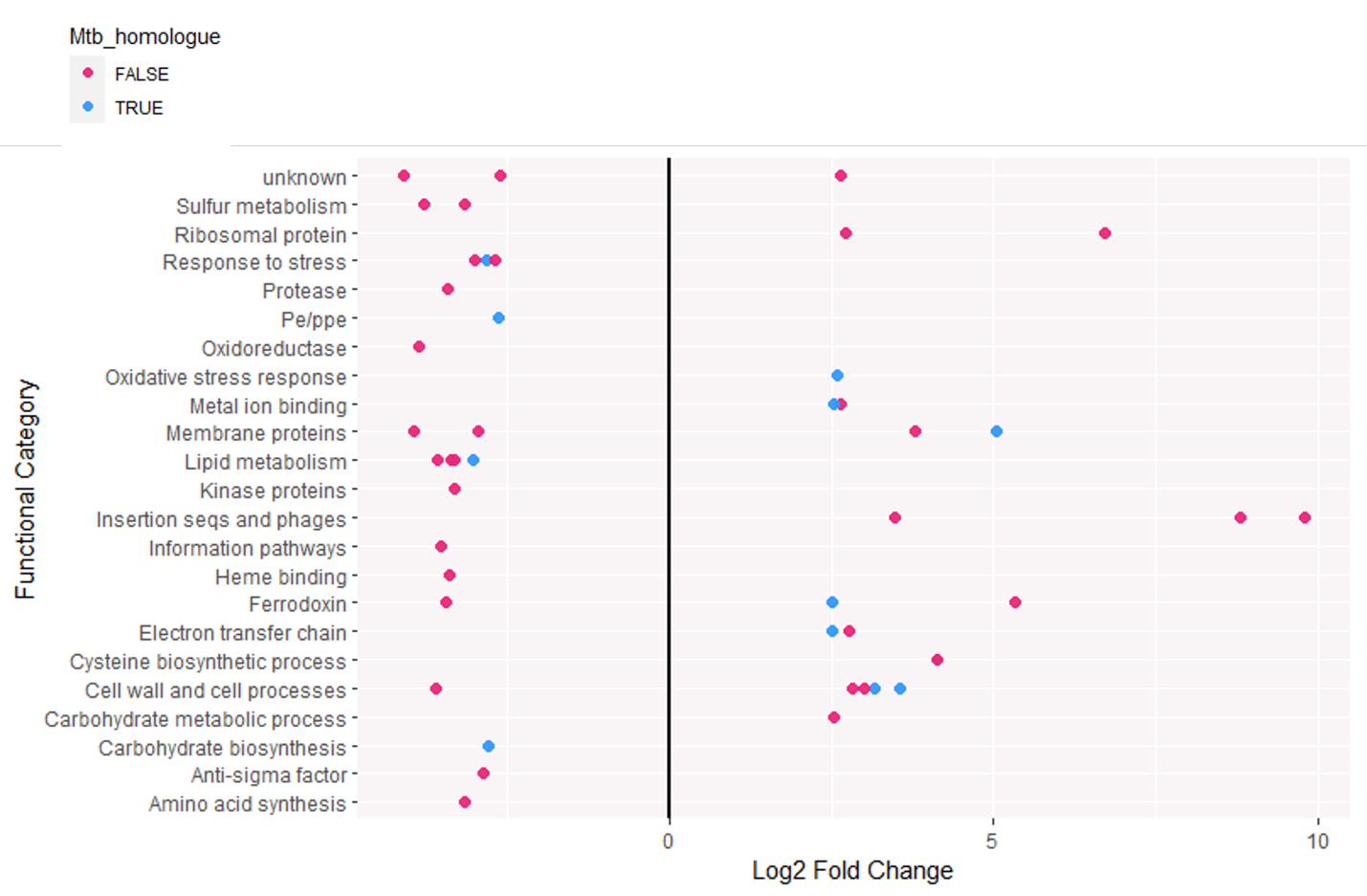


**Fig.S10**


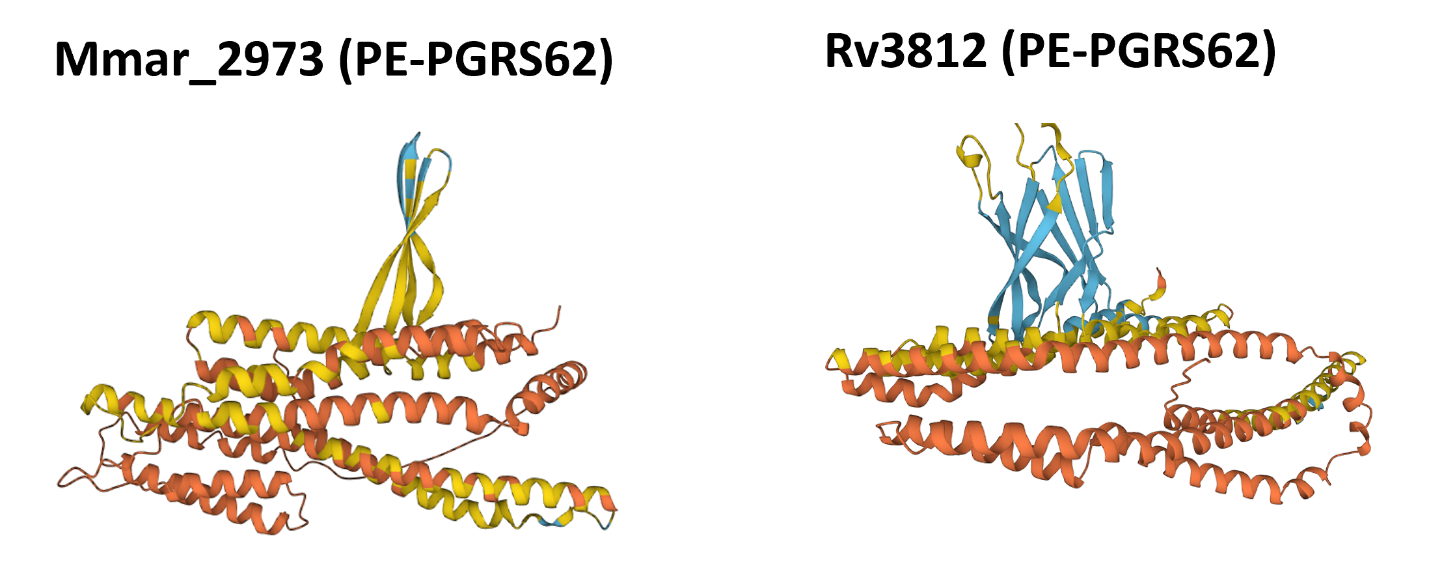


**Fig.S11**


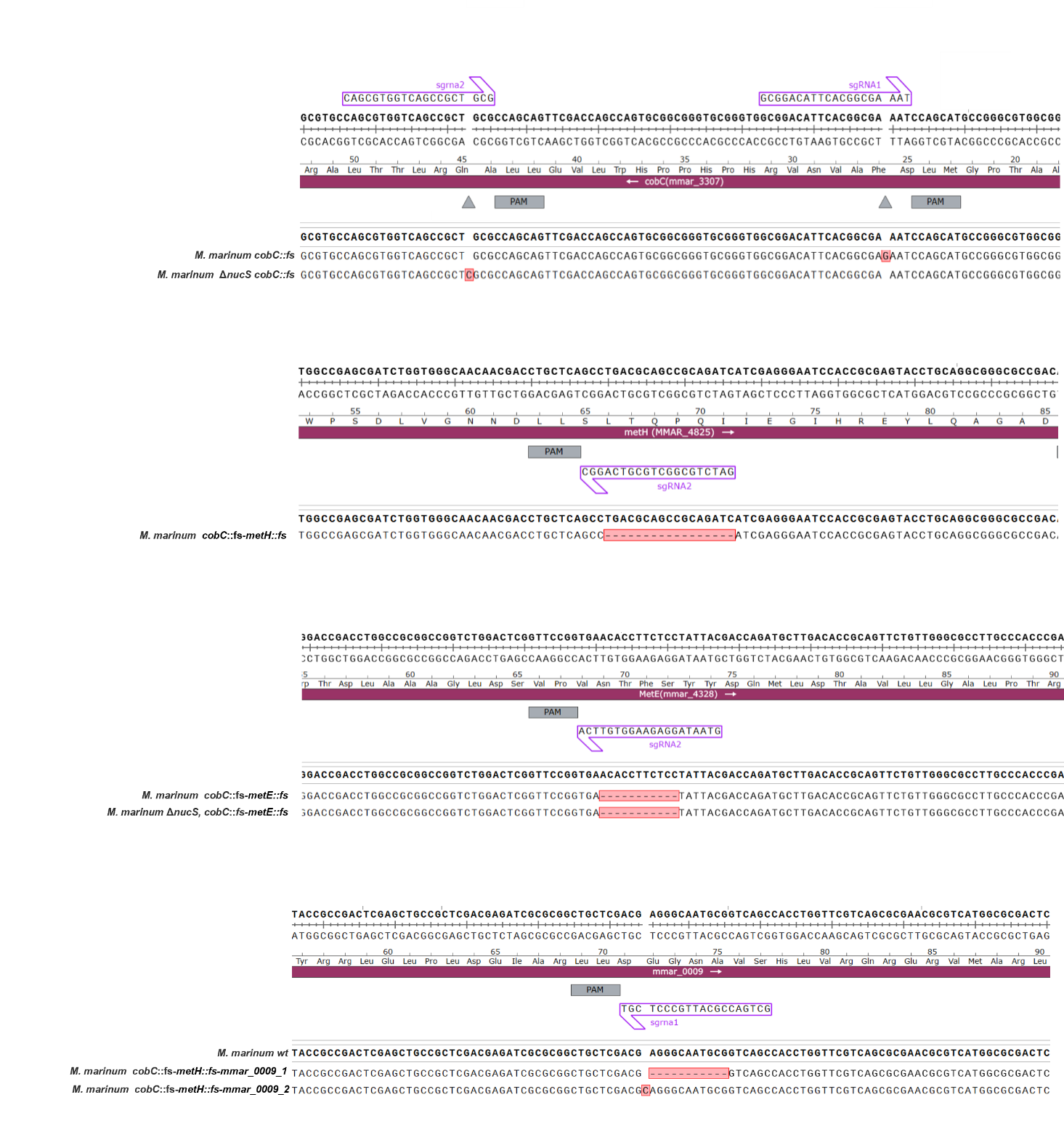


**SUPPLEMENTARY TABLES**

**Table S1**

| **Gene ID** | **Gene name** | **Orthologue H37RV** | **Type** | **Reference** | **Allele** | **Nucleotide change** | **Amino acid change** |
| --- | --- | --- | --- | --- | --- | --- | --- |
| **MMAR_0457** | **pseudogene** | - | Deletion | G | - | 465-466delG | Leu155fs |
| **MMAR_1593** | ***pe-pgrs* pseudogene** | - | Deletion | G | - | 2045-2046delG | Arg682fs |
| **MMAR_1594** | ***pe-pgrs*** | - | MNV | CA | AC | 4093CA>AC | Gly1365Thr |
| **MMAR_1927** | **hypothetical** | Rv2781c | Insertion | - | G | 524-525delG | Ala175fs |
| **MMAR_3097** | ***nrp*** |  | SNV | T | A | 2257T>A | Tyr853Asn |
| **MMAR_3307** | ***cobC*** | Rv2231c | Insertion | - | G | - | - |
| **MMAR_4328** | ***metE*** | Rv1133c | Deletion | ACACCTTCTCC | - | - | Val68fs |
| **MMAR_5443** | ***eccA1*** | Rv3868 | SNV | C | G | 1158C>G | Ile386Met |
| **MMAR_5555** | ***pe-pgrs*** | Rv3595c | Insertion | - | T | 840insT | Gly280fs |

| **Gene *M. marinum*** | **Gene name** | **Log₂ fold change** | **Functional category** | **Mtb homolog** |
| --- | --- | --- | --- | --- |
| **MMAR_5548** |  | -4.0861 | unknown |  |
| **MMAR_3049** | ***mmpS4_1*** | -3.91504 | Membrane proteins |  |
| **MMAR_3502** |  | -3.84792 | Oxidoreductase |  |
| **MMAR_0913** | ***sulP*** | -3.76299 | Sulfur metabolism |  |
| **MMAR_2074** |  | -3.57506 | Cell wall and cell processes |  |
| **MMAR_3406** |  | -3.56224 | Lipid metabolism |  |
| **MMAR_0944** |  | -3.50014 | Information pathways |  |
| **MMAR_2994** | ***fdxA_2*** | -3.43869 | Ferrodoxin |  |
| **MMAR_0916** |  | -3.41045 | Protease |  |
| **MMAR_2554** |  | -3.37877 | Heme binding |  |
| **MMAR_2071** | ***fadD11_1*** | -3.36413 | Lipid metabolism |  |
| **MMAR_2408** | ***pknF_2*** | -3.29712 | Kinase proteins |  |
| **MMAR_3488** | ***acs_1*** | -3.28858 | Lipid metabolism |  |
| **MMAR_3407** | ***gltD_1*** | -3.15664 | Amino acid synthesis |  |
| **MMAR_0914** | ***sulP_1*** | -3.15172 | Sulfur metabolism |  |
| **MMAR_1519** |  | -3.01017 | Lipid metabolism | Rv3130c |
| **MMAR_4922** |  | -2.99726 | Response to stress |  |
| **MMAR_2870** |  | -2.93172 | Membrane proteins |  |
| **MMAR_2847** |  | -2.86845 | Anti-sigma factor |  |
| **MMAR_3484** | ***hspX_1*** | -2.79348 | Response to stress | Rv2031c |
| **MMAR_2257** | ***otsB1*** | -2.77507 | Carbohydrate biosynthesis | Rv2006c |
| **MMAR_3478** |  | -2.68015 | Response to stress |  |
| **MMAR_2973** |  | -2.61697 | Pe/ppe | Rv3812 |
| **MMAR_5360** |  | -2.60282 | unknown |  |
| **MMAR_1522** |  | -2.40161 | Oxidoreductase | Rv3127 |
| **MMAR_3475** |  | -2.3748 | Oxidoreductase |  |
| **MMAR_4110** |  | -2.29306 | Intermediary metabolism and respiration |  |
| **MMAR_2685** | ***PPE30*** | -2.24375 | Pe/ppe |  |
| **MMAR_1403** |  | -2.22289 | Insertion seqs and phages |  |
| **MMAR_2620** |  | -2.21726 | Conserved hypotheticals |  |
| **MMAR_3155** |  | -2.16692 | Ferrodoxin |  |
| **MMAR_4287** |  | -2.14764 | Pe/ppe |  |
| **MMAR_2461** |  | -2.08944 | Pe/ppe |  |
| **MMAR_3516** | ***alkB_1*** | -2.04346 | Lipid metabolism |  |
| **MMAR_5272** | ***acrA1_1*** | -2.0289 | Lipid metabolism |  |
| **MMAR_1549** |  | -2.0192 | Lipid metabolism |  |
| **MMAR_2553** |  | -2.00824 | Cell wall and cell processes |  |
| **MMAR_1515** |  | -2.00007 | Response to stress | Rv3134c |
| **MMAR_5548** |  | -4.0861 | unknown |  |
| **MMAR_3049** | ***mmpS4_1*** | -3.91504 | Membrane proteins |  |
| **MMAR_3502** |  | -3.84792 | Oxidoreductase |  |
| **MMAR_0913** | ***sulP*** | -3.76299 | Sulfur metabolism |  |
| **MMAR_2074** |  | -3.57506 | Cell wall and cell processes |  |

**Table S2**

| **Gene *M. marinum*** | **Gene name** | **Log₂ fold change** | **Functional category** | **Mtb homolog** |
| --- | --- | --- | --- | --- |
| **MMAR_1445** |  | 9.78 | Insertion seqs and phages |  |
| **MMAR_1407** |  | 8.79 | Insertion seqs and phages |  |
| **MMAR_1086** | ***rpmj*** | 6.70 | Ribosomal protein |  |
| **MMAR_3421** | ***fdxc_1*** | 5.32 | Ferrodoxin |  |
| **MMAR_4345** |  | 5.04 | Membrane proteins | Rv115 |
| **MMAR_3417** | ***cysh_1*** | 4.13 | Cysteine biosynthetic process | Rv2392 |
| **MMAR_3416** | ***nira*** | 4.13 | Cysteine biosynthetic process |  |
| **MMAR_4903** |  | 3.78 | Membrane proteins |  |
| **MMAR_1928** | ***ald*** | 3.55 | Cell wall and cell processes | Rv2780 |
| **MMAR_3463** |  | 3.48 | Insertion seqs and phages |  |
| **MMAR_4665** | ***rpfa*** | 3.17 | Cell wall and cell processes | Rv0867c |
| **MMAR_4609** |  | 2.99 | Cell wall and cell processes |  |
| **MMAR_0275** | ***gcpe_2*** | 2.81 | Cell wall and cell processes |  |
| **MMAR_3739** |  | 2.77 | Electron transfer chain |  |
| **MMAR_2935** |  | 2.71 | Ribosomal protein |  |
| **MMAR_0233** |  | 2.65 | Metal ion binding |  |
| **MMAR_2668** |  | 2.64 | unknown |  |
| **MMAR_2755** | ***ahpc*** | 2.58 | Oxidative stress response | Rv2428 |
| **MMAR_3418** |  | 2.53 | Metal ion binding | Rv2393 |
| **MMAR_2753** | ***tres_1*** | 2.53 | Carbohydrate metabolic process |  |
| **MMAR_2080** | ***fdxa_1*** | 2.51 | Ferrodoxin | Rv2007c |
| **MMAR_2728** | ***ndh*** | 2.51 | Electron transfer chain | Rv1854c |
| **MMAR_4768** | ***cysk2*** | 2.49 | Cysteine biosynthetic process | Rv0848 |
| **MMAR_2754** | ***ahpd*** | 2.46 | Oxidative stress response | Rv2429 |
| **MMAR_2669** | ***ppe25*** | 2.45 | Pe/ppe |  |
| **MMAR_1365** | ***whib7*** | 2.33 | Whib-like protein | Rv3197A |
| **MMAR_4750** | ***dauA*** | 2.30 | Cysteine biosynthetic process |  |
| **MMAR_2138** |  | 2.27 | Methyltransferase | Rv2437 |
| **MMAR_3743** | ***mmps5_1*** | 2.23 | Membrane proteins |  |
| **MMAR_0151** | ***eis*** | 2.21 | Virulence, detoxification, adaptation |  |
| **MMAR_0792** | ***icl*** | 2.21 | Intermediary metabolism and respiration | Rv0467 |
| **MMAR_3462** |  | 2.18 | Insertion seqs and phages |  |
| **MMAR_2595** |  | 2.17 | unknown |  |
| **MMAR_4767** |  | 2.12 | Membrane proteins | Rv0849 |
| **MMAR_0335** |  | 2.09 | Electron transfer chain |  |
| **MMAR_2670** | ***pe19_1*** | 2.08 | Pe/ppe | Rv1788 |
| **MMAR_3443** | ***ppe61_1*** | 2.07 | Pe/ppe | Rv3532 |
| **MMAR_4413** |  | 2.06 | Electron transfer chain |  |
| **MMAR_2742** | ***xfp*** | 2.04 | Carbohydrate metabolic process |  |
| **MMAR_4904** |  | 2.03 | Cell wall and cell processes | Rv0784 |
| **MMAR_2872** |  | 2.03 | Membrane proteins |  |
| **MMAR_1132** | ***Whib3*** | 2.03 | Whib-like protein |  |
| **MMAR_4875** |  | 2.01 | Transcription regulator-repressor | Rv0968 |

**Table S3**

**Table S4**

| **Strain** | **Characteristics** | **Origin** | **Reference** |
| --- | --- | --- | --- |
| *M. marinum* WT | wild type | M^USA^ | ^55^ |
| *M. marinum* *cobC*::fs | *mmar_3307*:76insG, *pCRISPRx-Sth1-Cas9-L5* (^51^), sgRNA1, kanR, integrative | *M. marinum* WT | This study |
| *M. marinum* *cobC*::fs-*metH*::fs | *mmar_4825*:196-213del, *pCRISPRx-Sth1-Cas9-L5*, hygR, integrative | *M. marinum* *cobC*::fs | This study |
| *M. marinum* *cobC*::fs-*metH*::fs Tn library 100k mutants | Tn library | *M. marinum* *cobC*::fs-*metH*::fs | This study |
| *M. marinum* *cobC*::fs-*metH*::fs-*mmar_0009*::fs -214insT | *mmar_0009*:214insT, *pCRISPRx-Sth1-Cas9-L5*, sgRNA1, kanR, integrative | *M. marinum* *cobC*::fs-*metH*::fs | This study |
| *M. marinum* *cobC*::fs-*metH*::fs-*mmar_0009*::fs -217-230del | *mmar_0009*:217-230del, *pCRISPRx-Sth1-Cas9-L5*, sgRNA1, kanR, integrative | *M. marinum* *cobC*::fs-*metH*::fs | This study |
| *M. marinum* *cobC*::fs, *metE*::fs | *mmar_4328*:205-216del, *pCRISPRx-Sth1-Cas9-L5*, sgRNA1, hygR, integrative | *M. marinum* *cobC*::fs.*mmar_3307* | This study |
| *M. marinum* Δ*nucS* | Δ*nucS,* Δ*mmar_4077* | *M. marinum* WT | This study |
| *M. marinum* Δ*nucS,* *cobC*::fs | *mmar_3307:*133insC, *pCRISPRx-Sth1-Cas9-L5*, sgRNA2, kanR, integrative | *M. marinum* Δ*nucS cobC*::fs | This study |
| *M. marinum* Δ*nucS,* *cobC*::fs, *metE*::fs | *mmar_3307*:133insC-*mmar_4328*:205-216del, *pCRISPRx-Sth1-Cas9-L5*, sgRNA1, hygR, integrative | *M. marinum* Δ*nucS* | This study |
| *M. marinum* Δ*nucS,* *cobC*::fs, *metE*::fs-selected | selected in gradient plate | *M. marinum* Δ*nucS* *cobC*::fs-*metE*::fs | This study |
| *M. marinum* *papA5*::fs | *mmar_1768*:129delC, *pCRISPRx-Sth1-Cas9-L5*, sgRNA1, kanR, integrative | *M. marinum* WT | This study |
| *M. marinum* *ppsE*::fs | *mmar_1772*:1522insA, *pCRISPRx-Sth1-Cas9-L5*, sgRNA1, kanR, integrative | *M. marinum* WT | This study |

**Table S5**

| **Plasmid name** | **Characteristics** | **Reference** |
| --- | --- | --- |
| *piniB4-mcherry* | *promoter iniB4 region (mmar_0614-mmar_0616), mcherry, hygR, replicative* | ^15^ |
| *pMN437r* | *psmyc promoter, tdtomato, hygR, replicative* | ^53^ |
| *pMN016* | *pMN016 (MspA), psmyc promoter, hygR, replicative* | ^56^ |
| *pCRISPRx-Sth1-Cas9-L5* | *pLJR962, Sth1Cas9*, *kanR/hygR* versions, integrative L5 | ^51^ |
| *pLJR962* | *pLJR962, Sth1dCas9*, kanR/hygR versions, integrative L5 | ^30^ |
| *pSMT3-mmar0009* | *hsp60-promoter, mmar0009, hygR, replicative* | ^This work^ |
| *pMN016-metH* | *hsp60-promoter, rv2124c (metH), hygR, replicative* | ^This work^ |

| **Oligonucleotide** | **Sequence of sgRNA** | **PAM sequence** | **Fold repression ^30^** | **Forward primer 5'-3'** | **Reverse primer 5'-3'** |
| --- | --- | --- | --- | --- | --- |
| cobC-sgRNA1 | GCGGACATTCACGGCGAAAT | AGCAT | - | GGGAGCGGACATTCACGGCGAAAT | AAACATTTCGCCGTGAATGTCCGC |
| cobC-sgRNA2 | CAGCGTGGTCAGCCGCTGCG | AGCAG | - | GGGACAGCGTGGTCAGCCGCTGCG | AAACCGCAGCGGCTGACCACGCTG |
| metH-sgRNA1 | GATCTGCGGCTGCGTCAGGC | AGCAG | - | GGGAGATCTGCGGCTGCGTCAGGC | AAACGCCTGACGCAGCCGCAGATC |
| metE-sgRNA1 | GTAATAGGAGAAGGTGTTCA | GGAAC | - | GGGAGTAATAGGAGAAGGTGTTCA | AAACTGAACACCTTCTCCTATTAC |
| mmar_0009-sgRNA1 | GCTGACCGCATTGCCCTCGT | AGCAG | - | GGGAGCTGACCGCATTGCCCTCGT | AAACACGAGGGCAATGCGGTCAGC |
| papa5-sgRNA1 | GTCGGTGGCAATCGAGCAGGC | AGAAG | - | GGGAGTCGGTGGCAATCGAGCAGGC | AAACGCCTGCTCGATTGCCACCGAC |
| ppsC-sgRNA1 | GCCGCCGTCGTCAGCGACCGT | AGAAG | - | GGGAGCCGCCGTCGTCAGCGACCGT | AAACACGGTCGCTGACGACGGCGGC |
| mmar_2959-sgRNA | AGGGCGAACGGTGCAGCCAT | AGAAC | 120.5 | GGGAAGGGCGAACGGTGCAGCCAT | AAACATGGCTGCACCGTTCGCCCT |
| mmar_0009-sgRNA | GAGTCGGCCGGCAGCCCAGC | GGAAG | 145.2 | GGGAGAGTCGGCCGGCAGCCCAGC | AAACGCTGGGCTGCCGGCCGACTC |
| mmar_3798-sgRNA | GTCGAAATCCGCCACCCCGT | AGAAG | 216.7 | GGGAGTCGAAATCCGCCACCCCGT | AAACACGGGGTGGCGGATTTCGAC |
| mmar_1104-sgRNA | GTTCATGCGGCCGATGAACGCCT | AGCAG | 42.2 | GGGAGTTCATGCGGCCGATGAACGCCT | AAACAGGCGTTCATCGGCCGCATGAAC |
| mmar_4541-sgRNA | GATCGGCCGGTAAGGATGCT | GGAAG | 145.2 | GGGAGATCGGCCGGTAAGGATGCT | AAACAGCATCCTTACCGGCCGATC |

**Table S6**

**Table S7**

| **Oligonucleotide** | **Sequence 5'-3'** |
| --- | --- |
| NucS_KO_LF | TTTTTTTTGCATAAATTGCGTCGAAGACCACCAGGTCTC |
| NucS_KO_LR | TTTTTTTTGCATTTCTTGCCGTAGTCGACGGTGCATTGA |
| NucS_KO_RF | TTTTTTTTGCATAGATTGCTGTTCTGAGTGCCGCGATTA |
| NucS_KO_RR | TTTTTTTTGCATCTTTTGCATGCCGGACAGACACATCTT |
| Mmar0009-FW  Mmar0009-RV  MetH-FW | CGGAGGAATCACGCTAGCGTGGACGGACTCACCGTGGG  GTCGTACGGGTAACTAGTTCAGCCGCCGGTCCGGTTCG  AGCACGATCCGCATGCTTAATTAAGGGAGAACGTGACTGCGGCCGACAAGCA |
| MetH-RV | TCGAGGTCGACGGTATCGATTTAAACGTTGAAGTACTTGG |
